# SteatoNet: The First Integrated Human Metabolic Model with Multi-layered Regulation to Investigate Liver-Associated Pathologies

**DOI:** 10.1371/journal.pcbi.1003993

**Published:** 2014-12-11

**Authors:** Adviti Naik, Damjana Rozman, Aleš Belič

**Affiliations:** 1 Faculty of Computer Sciences and Informatics, University of Ljubljana, Ljubljana, Slovenia; 2 Faculty of Electrical Engineering, University of Ljubljana, Ljubljana, Slovenia; 3 Centre for Functional Genomics and Bio-Chips, Faculty of Medicine, University of Ljubljana, Ljubljana, Slovenia; The Pennsylvania State University, United States of America

## Abstract

Current state-of-the-art mathematical models to investigate complex biological processes, in particular liver-associated pathologies, have limited expansiveness, flexibility, representation of integrated regulation and rely on the availability of detailed kinetic data. We generated the SteatoNet, a multi-pathway, multi-tissue model and *in silico* platform to investigate hepatic metabolism and its associated deregulations. SteatoNet is based on object-oriented modelling, an approach most commonly applied in automotive and process industries, whereby individual objects correspond to functional entities. Objects were compiled to feature two novel hepatic modelling aspects: the interaction of hepatic metabolic pathways with extra-hepatic tissues and the inclusion of transcriptional and post-transcriptional regulation. SteatoNet identification at normalised steady state circumvents the need for constraining kinetic parameters. Validation and identification of flux disturbances that have been proven experimentally in liver patients and animal models highlights the ability of SteatoNet to effectively describe biological behaviour. SteatoNet identifies crucial pathway branches (transport of glucose, lipids and ketone bodies) where changes in flux distribution drive the healthy liver towards hepatic steatosis, the primary stage of non-alcoholic fatty liver disease. Cholesterol metabolism and its transcription regulators are highlighted as novel steatosis factors. SteatoNet thus serves as an intuitive *in silico* platform to identify systemic changes associated with complex hepatic metabolic disorders.

## Author Summary

In this article we present SteatoNet, the most comprehensive computational network of hepatic metabolism and the interaction of the liver with extra-hepatic tissues. Generation of the SteatoNet involved the application of engineering strategies to resolve prevalent drawbacks in biological modelling and thus effectively understand basic biology. SteatoNet does not require detailed kinetic parameters, behaves representatively of biological observations and portrays systemic interactions. SteatoNet is simple and flexible, which is an important advantage over current computational models of complex metabolic disorders associated with the liver. To demonstrate the utility of SteatoNet as a hypotheses-generation tool, we studied candidate mechanisms of hepatic fat accumulation, the key characteristic of non-alcoholic fatty liver disease (NAFLD), a common but poorly understood metabolic disorder influenced by genetic and lifestyle factors. Our data describe NAFLD as a network disease, with deregulated pathways such as inter-tissue transport of glucose, lipid and ketone bodies, cholesterol metabolism and “stiff” focal points in the fat tissue. These results indicate the involvement of systemic metabolic deregulations in the transformation of healthy to fatty liver in NAFLD. The SteatoNet highlights the utility of engineering approaches in systems biology to aid in solving complex biological questions.

## Introduction

Mathematical modelling provides an intuitive tool to investigate complex diseases that display multiple causal mechanisms and a complex pathobiology. In particular, liver-associated pathologies that have a high prevalence in Europe and other Western populations face poor prognosis, and the lack of adequate molecular understanding and management [1]. Hence, novel interdisciplinary strategies to comprehend the pathogenesis are required to quicken the pace of identifying suitable treatment regimens and monitor disease progression.

Mathematical models describing hepatic metabolic pathways have been constructed, including models of cholesterol synthesis [2], [3], glucose and lipid metabolism [4], [5] etc. HepatoNet1 that was constructed as a part of the Virtual Liver Network is a comprehensive reconstruction of a human hepatocyte [6], [7] which was further extended by a recent genome-scale metabolic model, iHepatocytes2322 describing the lipid metabolic pathways in detail [8]. While these extensive hepatocyte-specific models have immense potential to investigate liver functions, they may be less informative to study the aetiology of complex diseases, where deregulations occur in multiple tissues. An additional drawback of current metabolic models is the difficulty in simultaneously integrating metabolic pathways with both gene expression and signalling networks [9]. Thus, the robustness and genotype-phenotype correlation in these models is notably compromised. Moreover, a general hurdle for large computational models is parameter estimation since kinetic constants derived from *in vitro* experiments are at present poorly documented, display variability and are frequently incompatible with molecular behaviour *in vivo* [10].

Taking into consideration the currently prevalent drawbacks, we describe the SteatoNet, the first multi-pathway and multi-tissue model including key hepatic metabolic pathways, their interaction with extra-hepatic tissues and hierarchical feedback regulation at the gene expression and signal transduction levels. The kinetic parameters of the network are computed for a specified normalised steady state by utilizing user-defined values for the reversibility of reactions, the distribution of fluxes at pathway branches, and the metabolic influx into the network. Thus, the challenging task of accurately selecting kinetic parameters for large metabolic networks is bypassed. The estimated parameters are semi-quantitative and provide insights into the global system behaviour rather than accurate estimations of model variables.

In order to illustrate the utility of SteatoNet in investigating liver-associated pathologies, we describe a model analysis to identify candidate mediators involved in the initiation of non-alcoholic fatty liver disease (NAFLD). NAFLD, the hepatic manifestation of the metabolic syndrome, is the most common chronic liver disease in western populations with a prevalence of 25–30% [11]. The complexity and poor understanding of the NAFLD spectrum is emphasised by the small number of associated genetic variants identified by genome wide association studies [12]. Numerous metabolic pathways are involved in NAFLD pathogenesis (cell development, inflammation, fibrosis, endoplasmic reticulum stress, lipid and glucose metabolism, etc.), in addition to environmental factors and aberrant xenobiotic metabolism [13]. Thus, its multifactorial nature suggests that NAFLD is better described as a “network” disease instead of the currently accepted “two” or “three hit” disease hypothesis [14], [15]. SteatoNet analysis undertakes an engineering solution to provide evidence for the systemic multi-tissue characteristic of NAFLD initiation and highlights the utility of multi-tissue models that include regulatory aspects to investigate complex diseases.

## Results

**Figure 1:**
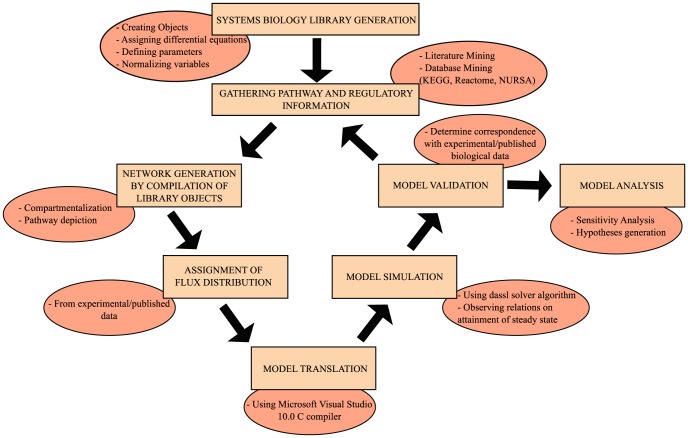
Summary of SteatoNet modelling workflow.

**Figure 2:**
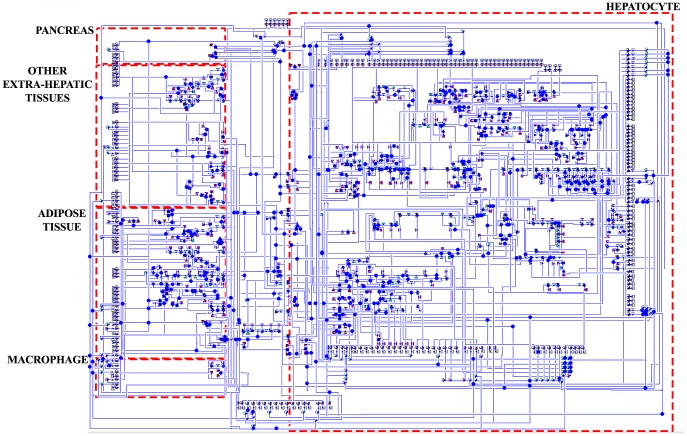
SteatoNet metabolic network. The key metabolic pathways and their regulation by hormones, adipokines and transcriptional and post-translational regulatory factors are represented in the hepatic, adipose, macrophage, peripheral tissue and pancreatic compartments with inter-tissue connectivity *via* the blood. The SteatoNet consists of 194 reactions with 159 metabolites, 224 enzymes and 31 non-enzymatic regulatory proteins.

**Figure 3:**
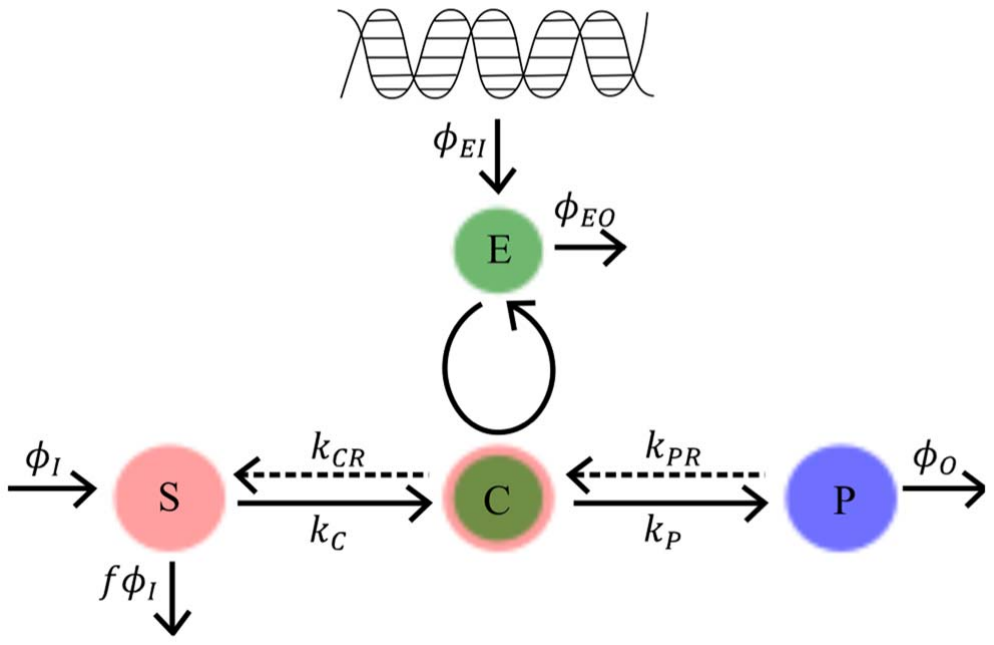
Dynamics of enzymatic reaction according to the Michaelis-Menten kinetic formalism. *S*, *E*, *C* and *P* denote the concentrations of the Substrate, Enzyme, substrate-enzyme Complex and Product respectively, *k_C_* and *k_P_* denote the rate constants of complex formation and product formation respectively, *k_CR_* and *k_PR_* the reverse reaction rate constants of complex dissociation into the enzyme and substrate and product reversibility to complex, respectively. *φ_I_* corresponds to the substrate influx, *φ_O_* to the product efflux, *φ_EI_* to the influx of enzyme, *φ_EO_* to the degradation of enzyme and *f* denotes the distribution of the total metabolic substrate flux into alternative pathways.

A systems biology library of objects corresponding to biological entities was utilised to compile the SteatoNet (Steatosis Network) in a systematic workflow (Fig. 1) to form a closed multi-pathway metabolic network (Fig. 2). The dynamics of each reaction in the SteatoNet (Fig. 3) is described by a set of differential algebraic equations (DAEs). The novelty in the approach utilized to generate SteatoNet is the definition of model parameters as a mathematical formalism based on reaction reversibility *r*, the distribution of the metabolic influx *f* into alternative pathways, the total influx *φ_I_* and the ratio between bound and free enzyme, *w*. This methodology transforms classical Michaelis-Menten kinetic parameters into a notation that differentiates static (*r*, *f*) and kinetic parameters (*w*, *φ_I_*). The model notation used in SteatoNet reduces the number of model parameters that must be derived from data or prior knowledge. In the presented model, 1046 or 25% of the total model parameters that describe the network must be manually set, the rest are calculated from the steady-state relations. Table 1 summarizes the model structure statistics.

**Table 1:** Model structure statistics.

**Variables and equations**	9270
**States and differential equations**	908
**Parameters**	5334
*bio-chemical pathway description*	4142
arbitrary set (independent)	1795
flux distribution*	116
reaction reversibility	195
free-bound enzyme ratio	195
gene expression control	1289
stoichiometric (independent)	1301
set at initialization (dependent)	1046
*simulation purpose* **	1192

*- data from Vo *et al*, 2006,

**-parameters included in the model to simulate perturbations of the system.

The SteatoNet comprises 194 reactions with 159 metabolites, 224 enzymes and 31 regulatory proteins. It includes the glucose, fatty acid, cholesterol and amino acid metabolic pathways, the inter-tissue transport of metabolites between the liver and extra-hepatic tissues and regulation at the transcriptional and post-translational levels by transcription factors, hormones, cytokines, adipokines and other regulatory factors. The hepatic compartment accounts for 70% of the SteatoNet model (104 metabolites, 184 proteins), the adipose compartment for 15% (24 metabolites, 40 proteins), other extra-hepatic tissues for 9% (14 metabolites, 24 proteins) and components in the blood and macrophage for the remaining 6% (17 metabolites, 7 proteins).

### SteatoNet validation

To validate the SteatoNet, we simulated metabolic conditions that have been well studied, including the response to fasting, the absence of stearoyl-CoA desaturase (SCD), a crucial lipogenic enzyme, overexpression of adiponectin, an insulin-sensitising anti-inflammatory cytokine released by the adipose and hepatic steatosis triggered by a high-fat diet, which can be subsided on treatment with a peroxisome proliferator-activated receptor alpha (PPARα) agonist. The fold-change in variable values in each simulation is with respect to their normalized value of 1.0 at the initial steady state. Hence, at steady state the observed changes are the net result of model perturbation. In the case of inconsistencies between experimental observations and model simulations, a series of simulations was implemented to identify the network components that display erroneous behaviour. Additional expert-based literature searches were performed to identify regulations that have been established experimentally but were absent in the network.

#### Fasting

Fasted state is characterised by low glucose and insulin levels and increased glucagon in the blood. Glucagon activates glycogen phosphorylase for the breakdown of stored glycogen to glucose and inhibits glycogen synthase. It upregulates gluconeogenesis by increasing the expression of phosphoenolpyruvate carboxykinase (PEPCK) and downregulates fatty acid synthesis by deactivating acetyl CoA carboxylase (ACC1). Fasting induces peroxisome proliferator-activated receptor alpha (PPARα), a nuclear receptor that regulates mitochondrial and peroxisomal fatty acid oxidation [16]. Additionally, fatty acid release from adipose tissue stores and oxidation are regulated by fatty acid-induced adipose factor (FIAF), which is upregulated during fasting [17].

**Figure 4:**
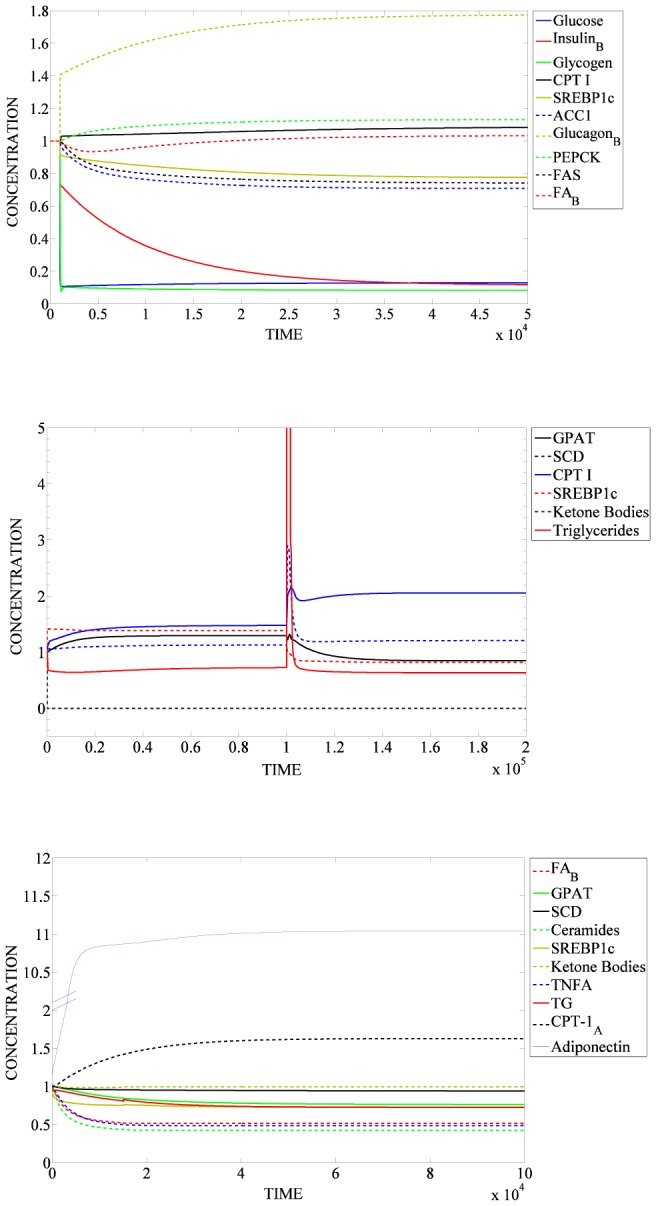
Validation of SteatoNet. a) Simulation of fasting condition, b) Simulation of stearoyl CoA desaturase (SCD) knockout. The lipogenic diet was simulated until time 1×10^5^ and the high fat diet was simulated between time 1×10^5^ and 2×10^5^. c) Simulation of adiponectin overexpression. Serum fatty acids (FA_B_), phosphoenolpyruvate carboxykinase (PEPCK), acetyl CoA carboxylase 1 (ACC1), fatty acid synthase (FAS), sterol regulatory element-binding protein-1c (SREBP-1c), carnitine palmitoyltrasnferase-1 (CPT1), glycerol-3-phophate acyltransferase (GPAT), hepatic triglycerides (TG), tumour necrosis factor alpha (TNFA), adipose carnitine palmitoyltransferase 1 (CPT1_A_).

To simulate fasting, the glucose influx into the network was reduced by 10-fold compared to the initial steady state. As illustrated in Fig. 4a and Table 2, the reduced glucose influx results in downregulation of serum insulin and glucose, hepatic glycogen stores, lipogenic enzymes (ACC1 and fatty acid synthase), sterol-regulatory element binding protein 1c (SREBP1c) and increased levels of serum glucagon, serum fatty acids, gluconeogenic enzyme PEPCK, the β-oxidation enzyme, carnitine acyl transferase-1 (CPT-1) and urea cycle enzymes. This corresponds accurately to the expected changes in the fasted state.

**Table 2:** Summary of SteatoNet validation conditions.

VALIDATION CONDITION	BIOLOGICAL OBSERVATIONS IN CONCORDANCE WITH STEATONET SIMULATIONS
Fasting	Downregulation of serum insulin and glucose
	Downregulation of hepatic glycogen stores
	Downregulation of lipogenic enzymes (ACC1 and FAS) and SREBP1c
	Upregulation of serum glucagon
	Upregulation of serum fatty acids
	Upregulation of gluconeogenic enzyme PEPCK
	Upregulation of the β-oxidation enzyme CPT-1.
Stearoyl CoA Desaturase knockout on a lipogenic diet (until time = 10^5^ units) and on a high-fat diet (time 10^5^ to 2×10^5^ units).	Downregulation of hepatic triglyceride accumulation on both diets
	Upregulation in CPT-1 and ketone bodies on both diets
	Upregulation of lipogenic enzyme GPAT and its transcriptional regulator SREBP-1c on lipogenic diet
	Downregulation of GPAT and SREBP-1c on a high-fat diet
Adiponectin Overexpression under fasting conditions	Downregulation of serum fatty acids
	Downregulation of hepatic triglyceride
	Downregulation of TNF-α
	Downregulation of ceramide,
	Downregulation of hepatic lipogenic genes SREBP1c, GPAT and SCD1
	Upregulation of adipose β-oxidation
PPAR-alpha agonism to improve hepatic steatosis	Downregulation of hepatic triglyceride
	Upregulation of fatty acid oxidation,
	Downregulation of plasma fatty acids
	Downregulation of plasma VLDL
	Downregulation of hepatic cholesterol
	Downregulation of LDL cholesterol

ACC1- Acetyl CoA Carboxylase 1, CPT-1- carnitine acyl transferase-1, FAS- Fatty acid synthase, GPAT- glycerol-3-phosphate acyltransferase, PEPCK- Phosphoenol pyruvate carboxykinase, SCD-1- Stearoyl CoA Desaturase 1, SREBP-1c- sterol-regulatory element binding protein 1c, TNF-α- Tumour necrosis factor alpha.

#### Stearoyl CoA knockout

Stearoyl CoA desaturase (SCD) is a rate-limiting delta-9 desaturase enzyme involved in *de novo* lipogenesis and lipid metabolism [18]. It catalyses the conversion of saturated fatty acids, in particular oleate (18∶1) and palmitoleate (16∶1), into monounsaturated fatty acids, which serve as the key building blocks of triglycerides, cholesterol esters and membrane phospholipids. The SCD-1 isoform is predominantly expressed in the liver and its expression is tightly regulated. *SCD−/−* mice fed a high fat diet display protection against diet-induced obesity and increased insulin sensitivity due to an upregulation of fatty acid oxidation and downregulation of lipogenesis [19]. Although a lipogenic (high-carbohydrate) diet increases the expression of genes involved in *de novo* lipogenesis, *SCD−/−* mice fed a lipogenic diet still display low levels of triglycerides, indicating the crucial role of SCD-1 in regulating triglyceride synthesis [20]. SCD-1 activity is also increased in NAFLD patients [21].

To simulate the SCD knockout condition, the rate of SCD enzyme degradation was increased by 1000-fold, resulting in SCD enzyme concentration approaching 0 (Fig. 4b). A lipogenic diet was simulated by increasing the glucose influx by 10-fold. The high-fat diet was simulated by increasing the influx of triglycerides (5-fold) and cholesterol (4-fold), and decreasing the glucose influx by 2.5-fold (fold-changes approximated from the composition of laboratory high fat diet and regular chow diet for mice). Fig. 4b and Table 2 illustrate the effects of SCD absence on a lipogenic diet until time = 10^5^ units and on a high-fat diet between time 10^5^ and 2×10^5^ units. Simulations of the response of triglyceride concentration, fatty acid oxidation and lipogenesis enzymes indicate a decrease in hepatic triglyceride accumulation and increase in CPT-1 and ketone bodies on both diets. While the lipogenic enzyme glycerol-3-phosphate acyltransferase (GPAT) and its transcriptional regulator SREBP-1c were upregulated by the lipogenic diet, their concentrations decreased on a high-fat diet, in concordance with experimental observations in rodent models. The spike in triglyceride concentration observed at time = 1×10^5^ units results from the switch between the lipogenic diet (high glucose) to the high-fat diet. Introduction of high fat diet causes a hepatic overload of fatty acids and triglyceride derived from the dietary source, however in the absence of SCD as simulated in Fig. 4b, this overload is short-lived as there is minimal *de novo* synthesis of triglycerides. Hence, excess lipids are either oxidized or distributed to maintain triglyceride homeostasis.

#### Adiponectin overexpression

Adiponectin is an adipose-secreted cytokine that correlates negatively with insulin resistance, plasma triglycerides and low-density lipoprotein (LDL) – cholesterol, hepatic fat content and progression to NASH in NAFLD patients [22], [23]. The beneficial impact of adiponectin on metabolism and insulin resistance is enforced by the ceramidase activity of the AdipoR1 and AdipoR2 receptors [24]. A 30% decrease in plasma fatty acids, upregulation in ketone bodies and fatty acid oxidation in the adipose tissue, and decreased expression of lipogenic genes was observed on fasting in adiponectin-overexpressing transgenic mice [25]. Under conditions of hyperleptinemia and adiponectin overexpression, white adipose cells display high levels of fat oxidation due to upregulation of oxidation genes such as peroxisome proliferator-activated receptor α, peroxisome proliferator-activated receptor-γ coactivator 1α, and uncoupling proteins [26]. Whilst adiponectin has insulin-sensitizing, anti-inflammatory and antilipogenic effects, tumour necrosis factor alpha (TNF-α) is thought to be a suppressor of adiponectin. Adiponectin-null mice display high levels of TNF-α mRNA and protein and diet-induced insulin resistance, thus explaining the phenomenon of increased TNF-α in obese populations [27].

To simulate the overexpression of adiponectin under fasting conditions, the rate of adiponectin degradation was decreased by 10-fold, resulting in increased levels of adiponectin, and the glucose influx was reduced by 10-fold. In concordance with experimental observations, increased adiponectin concentration results in downregulation of serum fatty acids, hepatic triglyceride, TNF-α, ceramide, hepatic lipogenic genes SREBP1c, GPAT and SCD1 and increased expression of adipose CPT-1, indicating an upregulation of β-oxidation in adipocytes (Fig. 4c and Table 2). The simulation did not indicate any changes in serum ketone body concentration.

#### Targeting peroxisome proliferator-activated receptor alpha

Fenofibrates, PPARα agonists, have been investigated in-depth for their beneficial effects on various features of the metabolic syndrome. Treatment with a specific PPARα agonist, Wy 14,634, in various mouse models with metabolic deregulations such as lipoatrophic diabetes [28], spontaneous fatty liver in the absence of obesity or T2D [29], NAFLD [30] and NASH [31] resulted in improved dyslipidaemia, insulin resistance and hepatic steatosis.

**Figure 5:**
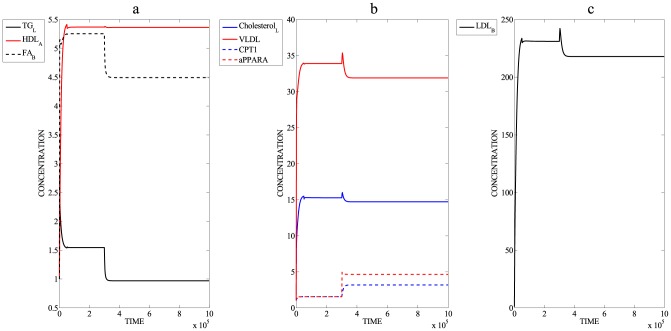
Peroxisome proliferator-activated receptor alpha (PPARα) activation in high fat diet- induced steatosis. Panels a–c illustrate the simulation of different variables in a background of hepatic steatosis induced by a high-fat diet (increased triglyceride and cholesterol influx, from time = 0) and subsequent treatment with a PPARα agonist (from time = 3×10^5^). a) Simulation of hepatic triglyceride (TG_L_), plasma high-density lipoprotein (HDL_B_) and serum fatty acids (FA_B_); b) Simulation of hepatic cholesterol (Cholesterol_L_), plasma very low-density lipoprotein (VLDL), carnitine palmitoyltransferase 1 (CPT1) and active proliferator-activated receptor alpha (aPPARα); c) Simulation of low-density lipoprotein (LDL_B_).

Fig. 5 panels a–c and Table 2 illustrate the SteatoNet simulation of different variables in a background of hepatic steatosis induced by a high-fat diet (from time = 0 units) and subsequent treatment with a PPARα agonist (from time = 3×10^5^ units). Simulation of a high fat diet by upregulating the triglyceride and cholesterol influx by 5-fold and 4-fold respectively, and downregulating the glucose influx by 2.5-fold results in hepatic triglycerides accumulation by 1.5-fold (Fig. 5a) and upregulation of plasma fatty acids (Fig. 5a), VLDL (Fig. 5b), hepatic cholesterol (Fig. 5b) and LDL (Fig. 5c). Under these conditions, treatment with fenofibrates i.e. PPARα activation (Fig. 5b, starting from time = 3×10^5^ units) diminishes hepatic triglyceride accumulation (Fig. 5a), upregulates fatty acid oxidation illustrated by CPT-1 (Fig. 5b), lowers plasma fatty acids (Fig. 5a) and VLDL (Fig. 5b) in addition to decreasing hepatic cholesterol (Fig. 5b) and LDL-cholesterol (Fig. 5c) with no effects on plasma HDL (Fig. 5a). These simulation results are in accordance with biological observations on treatment with PPARα agonists. The transient spike in hepatic triglyceride concentration in Fig. 5a results from the rapid influx of lipids when simulating a high fat diet; however, the net increase in triglyceride concentration after reaching the steady state is 1.5-fold.

In conclusion, model validation simulations are representative of biological observations and thus, the SteatoNet can be utilised as an *in silico* tool to assess liver-associated deregulations.

#### Identification of deregulations associated with NAFLD by flux distribution sensitivity analysis

To demonstrate the effectiveness of SteatoNet in investigating disorders related to hepatic metabolism, the model was subjected to sensitivity analysis to highlight candidate mechanisms that trigger hepatic accumulation of triglycerides, a key characteristic of NAFLD.

Considering the interconnected nature of metabolic networks, metabolic flux through a pathway and its distribution among sub-branches is a crucial parameter. Its value may depend upon flux entering the network, enzyme activity or concentration, and genetic variations. Extrapolating from the ‘network disease’ hypothesis for NAFLD, we determined the relevance of flux distributions at various SteatoNet pathway branches in triggering hepatic triglyceride accumulation under a high calorie diet (increased glucose and triglyceride influx).

**Figure 6:**
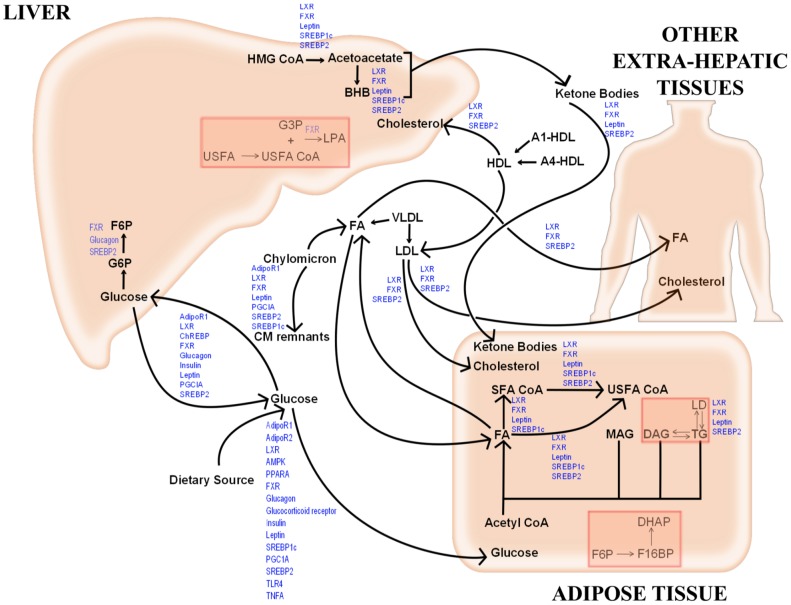
Hepatic triglyceride sensitivity network. The represented pathways highlight high impact branches that significantly influence hepatic triglyceride concentration. The branch-points in the red boxes indicate high impact branches with low tolerance to flux alterations. Pathway regulators (blue text) that are influenced by alterations in flux distribution are labelled at the corresponding high impact branch-points. AdipoR- Adiponectin Receptor, AMPK- Adenosine Monophosphate- activated Kinase, BHB- beta-Hydroxybutyrate, ChREBP- Carbohydrate Response Element Binding Protein, CM- Chylomicron, DAG- Diacylglycerol, DHAP- Dihydroxyacetone phosphate, FA- Fatty Acid pool, F6P- Fructose-6-phosphate, F16BP- Fructose-1,6-bisphosphate, FXR- Farnesoid×Receptor, G3P- Glycerol-3-Phosphate, G6P- Glucose-6-Phosphate, HDL- High Density Lipoprotein, HMG CoA- 3-Hydroxy 3-Methylglutaryl CoA, LD- Lipid Droplet, LDL- Low Density Lipoprotein, LPA- Lysophosphatidic Acid, LXR- Liver×Receptor, MAG- Monoacylglycerol, PGC1A- Peroxisome Proliferator- Activated Receptor Gamma Coactivator 1 alpha, PPARA/G- Peroxisome Proliferator- Activated Receptor Alpha/Gamma, SFA CoA- Saturated Fatty Acyl CoA, SREBP- Sterol Regulatory Element Binding Protein, TG- Triglyceride, TLR4- Toll-like Receptor 4, TNFA- Tumour Necrosis Factor Alpha, USFA CoA- Unsaturated Fatty Acyl CoA, VLDL- Very-Low Density Lipoprotein.

The branch-points were classified according to their concentration control coefficients with respect to hepatic triglycerides as ‘high’ (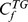
>1), ‘moderate’ (0.1≤
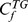
≤0.99) or ‘low’ (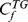
<0.1) impact. A high 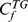 value (high impact) of a branch indicates that the metabolic flux distribution at this point in the network significantly influences the concentration of hepatic triglycerides. Additionally, several branch-points displayed low tolerance to flux changes i.e. incurred instability beyond a limited range of metabolic influx and were sub-classified as low tolerance branch-points. Table 3 lists branch-points that have a high impact on hepatic triglyceride concentration but low tolerance. We also determined the sensitivity of regulatory factors to alterations in flux distributions. Fig. 6 illustrates high impact branch-points that significantly influence hepatic triglyceride concentration, and their associated regulatory factors.

**Table 3:** Pathway branch-points with high 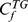 and low flux range tolerance.

PATHWAY BRANCH	FLUX RANGE	SENSITIVITY COEFFICIENTS
FA (+Gly-3-P) to LPA	Upto 10% of total flux into fatty acids	2.098
TG_A_ storage in LD_A_	20–30% of total flux into adipocyte triglycerides	1.224
TG_A_ to DAG_A_	50–60% of total flux into adipose triglycerides	2.781
F16BP_A_ to DHAP_A_	20–40% of total flux into F16BP_A_	1.12 to 8.81

DAG- Diacylglycerol, DHAP- Dihydroxyacetone Phosphate, F16BP- Fructose 1,6-Bisphosphate, FA- Fatty acids, Gly-3-P- Glycerol-3-phosphate, LD- Lipid droplets, LPA- Lysophosphatidic acid, TG- Triglycerides, _A_- adipose compartment.

**Figure 7:**
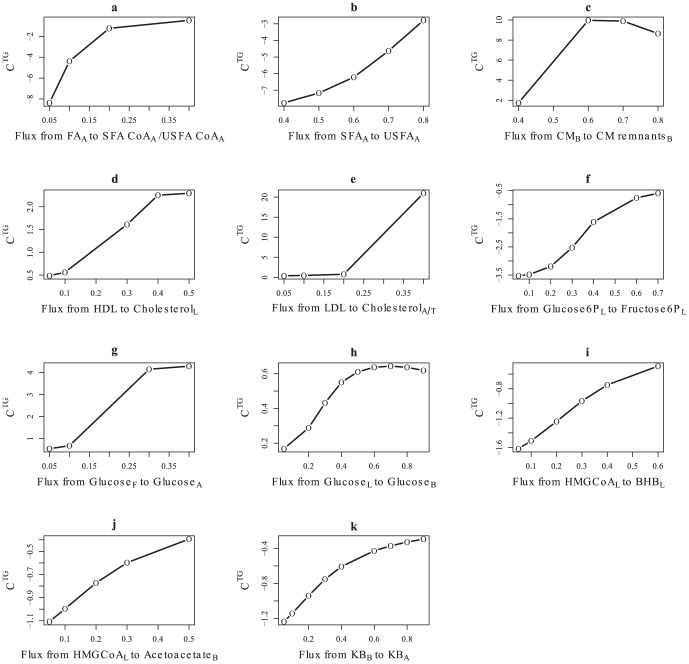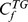 of branch-points in SteatoNet. Range of 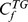 of a) activation of saturated (SFA) and unsaturated (USFA) fatty acids in adipose, b) desaturation of SFA to USFA in adipose, c) breakdown of chylomicron into chylomicron remnants, d) reverse cholesterol transport, e) LDL distribution to adipose and peripheral tissues, f) fructose-6-phosphate synthesis from glucose-6-phosphate, g) glucose transport to adipose, h) hepatic release of glucose into blood, i) β-hydroxybutyrate (BHB) synthesis from 3-hydroxy 3-methylglutaryl coenzyme A (HMG CoA), j) acetoacetate transport to blood, and k) uptake of ketone bodies (KB) by adipose.

The sensitivity analysis indicated that a range of values of *f* ensure correspondence to biological observations instead of a unique value in the majority of branch-points. This is in agreement with the underlying biology where a number of physiological steady states are possible. However, for a minority of branch points (Fig. 6, Fig. 7 and Table 3), the flux distribution parameter impacts model behavior significantly. For these cases, the value of *f* affects the variable values quantitatively only (degree of fold-change) but not qualitatively i.e. no branch-points were identified where a change in *f* altered the direction of fold-change in variable values. Interpretations from the SteatoNet are qualitative in nature; hence, the set/assumed values of *f* do not affect the conclusions drawn from the model simulations. However, in real biological systems, the values of *f* at the highlighted branch-points significantly impact the concentration of biological entities whereby even subtle fold-changes may result in phenotypic changes. The mentioned concentration control coefficients are estimates calculated from SteatoNet variables and have been presented to illustrate the relative degree of variation resulting from changes in flux distribution. Branch-points that significantly impact hepatic triglyceride concentration and their regulators are discussed in detail in the following section.

#### High impact branches (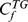
>1)

A majority of the branch-points that significantly impact hepatic triglyceride concentration occur in the inter-tissue transport of lipid, glucose and ketone bodies (Fig. 6) as discussed below, highlighting the crucial role of metabolite transport and redistribution in maintaining lipid homeostasis. Moreover, the relation between triglyceride concentration and flux alternations does not appear to be simply linear but is dynamic in nature, as indicated by the non-linear variations of 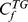 absolute values within the tested flux range (Fig. 7).

Fig. 6 highlights the enrichment of lipid metabolism amongst the pathways with a high impact on hepatic triglyceride concentration. These include activation and desaturation of adipose fatty acids; lipolysis in the adipocyte and transport of fatty acids between the blood, adipocyte and peripheral tissues; contribution of the diet (*via* chylomicrons) and very-low density lipoprotein (VLDL) breakdown to serum fatty acids; and transport of serum cholesterol to the liver (reverse cholesterol transport *via* high-density lipoprotein), adipose and other extra-hepatic tissues (Fig. 7a–7e).

An increased flux of adipose-derived fatty acids contributes to hepatic steatosis in NAFLD patients [32]. This observation is confirmed in our study whereby the negative 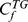 of the desaturation and activation of adipose fatty acids (Fig. 7a and 7b) indicates a negative correlation between adipose-specific lipogenesis/lipid storage and hepatic triglyceride concentration. Moreover, SteatoNet indicates that hepatic uptake of fatty acids from the blood moderately impacts triglyceride concentration and displays low tolerance (S1 Table), suggesting that only a limited range of fatty acid influx into the hepatocyte is tolerated for a healthy state.

Cholesterol accumulation-related lipotoxicity and oxidative stress, along with increased expression of genes involved in cholesterol synthesis, is a hallmark of obese insulin-resistant NAFLD and non-alcoholic steatohepatitis (NASH) rodent models and patients [33], [34]. In accordance with these observations, SteatoNet indicates that perturbations in reverse cholesterol transport and in cholesterol distribution between adipose and other extra-hepatic tissues critically influence hepatic steatosis (Fig. 6, Fig. 7d–7e). The 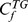 of extra-hepatic LDL distribution displays a positive correlation with hepatic triglyceride concentration (Fig. 7e), whereby moderate LDL fluxes to extra-hepatic tissues do not significantly affect triglyceride concentration. However, at a higher LDL flux, the hepatic triglyceride concentration increases exponentially. LDL-cholesterol transport to the liver is important for triglyceride-rich VLDL secretion [35]. Thus, a fine balance in cholesterol concentration is important to sustain VLDL secretion, whilst preventing steatosis or lipotoxicity. Hence, the role of cholesterol in NAFLD pathogenesis may commence at the early stage of steatosis, besides the commonly accepted notion of cholesterol-triggered lipotoxicity that prompts progression to NASH.

The exchange of glucose between the serum and liver, transport of glucose to the adipose tissue and contribution of dietary glucose to serum glucose level, display a significant influence on hepatic triglyceride concentration (Fig. 6, Fig. 7f–7h). SteatoNet indicates a negative correlation between hepatic triglyceride concentration and early glycolysis (Fig. 7f). This can be explained by the decreased activation of lipogenic factors ChREBP and SREBP-1c by glucose [36] and insulin [37] as a result of glucose catabolism. For the high calorie conditions tested here, glucose flux towards lipogenesis is limited due to extensive availability of fatty acids as substrates and hence, does not directly influence hepatic steatosis.

The synthesis and transport of acetoacetate and β-hydroxybutyrate (BHB) display a significant negative impact on hepatic triglyceride accumulation (Fig. 6, 7i–7k). A genetic screen in zebrafish highlighted a mutation in solute carrier family 16a, member 6a (Slc16a6a), a transporter of BHB, in diverting it as a substrate for lipogenesis, which resulted in hepatic triglyceride accumulation [38]. Serum levels of ketone bodies also indicate the extent of hepatic mitochondrial β-oxidation [39]. Thus, the synthesis and extra-hepatic transport of ketone bodies is an indicator of hepatic triglyceride concentrations, as confirmed by the SteatoNet sensitivity analysis.

#### High impact branches with low tolerance

Four pathway branches in the SteatoNet in particular display a significant influence on hepatic triglyceride concentration but low tolerance to variations in flux distribution (Table 3). Beyond a narrow permitted range of flux distribution values, the model incurred instability and was unable to reach a new steady state. Such high impact branches with low tolerance are specifically enriched in triglyceride metabolism, indicated by red boxes in Fig. 6, including formation of hepatic lysophosphatidic acid (LPA), the first step in triglyceride synthesis; adipose triglyceride storage in lipid droplets and breakdown into diacylglycerol (DAG); and adipose-specific synthesis of dihydroxyacetone phosphate (DHAP), a precursor for glycerol-3-phosphate involved in *de novo* lipogenesis (Fig. 6). These focal points in the network may play a key role in initiating NAFLD by destabilizing lipid homeostasis at even slightly increased metabolic flux. With the enrichment of low tolerance branches in the adipocytes (three out of four), SteatoNet confirms the role of adipose triglyceride storage/hydrolysis on hepatic lipid accumulation, which has been proven in several studies [40]–[42]. Although the production of adipose-specific DHAP from fructose-1,6-bisphosphate displays tolerance to a broader range of flux alterations (within 20% compared to 10% in the other three high impact branches, Table 3), there is a steep increase in the 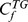 (from 1.12 to 8.81) at the upper limit of the tolerated flux range. This observation highlights the significantly increasing impact of adipose glucose metabolism on hepatic lipid accumulation. The low tolerance in the hepatic fatty acids distribution to form LPA is crucial due to its direct role in *de novo* lipogenesis. LPA is the substrate for the lysophosphatidic acid acyltransferase reaction catalysed by adiponutrin/PNPLA3 [43]. A polymorphism *rs738409 C/G*, encoding the gain-of-function *PNPLA3 I148M* variant consistently correlates to NAFLD in various populations [44]–[46]. The low tolerance of fatty acid distribution for LPA formation may act as a mechanism to ensure limited availability of substrate for triglyceride synthesis. A breach of this limited flux range may trigger hepatic triglyceride accumulation, especially in the presence of the *PNPLA3 I148M* polymorphism.

#### Regulators of high impact branches

Pathway branches that significantly influence hepatic triglyceride concentration were further investigated to identify regulatory factors that are also affected by flux alterations and hence, may indirectly but synergistically impact hepatic steatosis. Hence, we calculated the concentration control coefficients of these branches with respect to regulatory proteins in the SteatoNet. Fig. 6 illustrates the regulatory factors with high sensitivity to the respective pathway branch-points. The role of cholesterol metabolism in NAFLD pathogenesis is further emphasised by the network-wide sensitivity of FXR, a regulator of bile acid synthesis and excretion; LXR, a sterol-sensor; and SREBP2, a key regulator of cholesterol synthesis. The direct effect of these cholesterol metabolism-related transcription factors on hepatic triglyceride accumulation, the primary stage of NAFLD, under conditions of a high calorie diet further emphasizes the role of cholesterol in the early steatosis stage of NAFLD. FXR is recognised as a potential target for NAFLD treatment [47]. NAFLD patients display low FXR levels along with increased expression of LXR and SREBP1c, thus contributing to increased hepatic triglyceride synthesis [48].

Interestingly, SteatoNet indicates that transcription factors that are not directly involved in the regulation of a particular pathway can still be sensitive to flux distributions within the pathway e.g. the sensitivity of FXR and SREBP2 to branch-points in early hepatic glucose metabolism (Fig. 6). This can be explained by the interconnected nature of metabolic networks, as metabolites generated by one pathway are utilised as substrates by another pathway. Thus, the inter-dependency of pathways may simultaneously be controlled by a balance in various transcription factors.

## Discussion

As suggested by Lanpher *et al* [49], most complex diseases can be described as less severe cases of Mendelian inborn errors of metabolism (IEM) in which several pathways are subtly affected. The cumulative effect of genomic variations and environmental/dietary factors results in altered metabolic flux distributions and a broad spectrum of disease phenotypes. Thus, utilising a holistic approach to study disease-related networks can provide a clearer portrait of the systemic deregulations in complex diseases. It can also guide drug development strategies towards systems medicine and multi-targeting approaches.

In this article we present SteatoNet, a closed multi-compartmental metabolic network, which serves as an *in silico* platform to systematically investigate hepatic metabolic phenomena and associated deregulations. Validation of SteatoNet and identification of deregulated flux disturbances that have been proven experimentally in NAFLD patients or in animal models highlights the ability of SteatoNet to describe biological behaviour by steady-state analysis in the absence of experimentally determined kinetic parameters.

The problem of parameter estimation in complex models is a major limiting factor for accurate reconstructions of biological pathways. Models thus often involve multiple assumptions. Our approach bypasses this hurdle and underlines the importance of expansiveness and structural accuracy for representation of biological systems. This methodology is sufficient for qualitative modelling whereby the model is utilized to study particular biological phenomena and hypothesize/identify network-wide changes in response to particular disturbances. Although the method may not provide precise absolute values of the variables, it overcomes a major problem in biological modelling by bypassing the need to fix enzymatic parameters for an enormous number of enzymes in such large-scale multi-tissue models. The application of modelling techniques from machinery, based on object libraries and closed circuits, proves to be appropriate and efficient. The physiological state of an organism is a result of complex system-wide interactions and hence, analysing individual pathways in isolation leads to loss of information and inaccurate interpretations. Analysing the broad scheme of the ‘candidate’ disease network and then channelling efforts towards specific molecular interactions is a fundamental primary step in systems pathobiology.

A dominant and unique feature of SteatoNet is integration of the metabolic network with regulation at the gene expression and signal transduction levels, which contributes to its robustness and effective concurrence with biological behaviour. In spite of its limited quantitative predictive value resulting from bypassing kinetic parameters and integrating multiple types of complex information (signalling, gene expression, metabolic reactions), SteatoNet provides an effectively regulated system-wide virtual space for generating scientifically sound hypotheses. The omission of details in the model may have an important impact on the small-scale cell metabolism but is completely masked at a network-wide scale. The SteatoNet is thus the first integrated model of human metabolism that represents the multiple layers of metabolic regulation.

Experimental evidence has indicated the systemic nature of NAFLD pathogenesis, involving the role of the adipose tissue [50], skeletal muscles [51] intestine [52] and the heart [53], however, directed efforts to study NAFLD as a systemic condition were lacking. *iHepatocytes2322*, a comprehensive genome-wide network reconstruction of hepatocyte metabolism was utilised to analyse transcriptomic data from NASH patients and identify diagnostic biomarkers [8]. While this study identified the role of pathways such as serine metabolism in NASH, the model is inadequate to capture systemic metabolic changes in the organism, which is especially critical in the initial steatosis stage of NAFLD as observed from our study.

Sensitivity analysis of the SteatoNet was aimed to identify small changes in pathway fluxes that significantly influence hepatic triglyceride concentration. Such focal points in the network have a significant effect on NAFLD onset, as small disturbances in the metabolic network can result in large effects on triglyceride concentration. To-date, a limited number of polymorphisms have been associated with NAFLD populations indicating the absence of dominant genetic aberrations that are causal of this disorder. Taking into consideration the complexity of NAFLD, it can be expected that the disease is initiated as a result of multiple subtle changes in the metabolic homeostasis ultimately resulting in hepatic steatosis.

We provide evidence that NAFLD is not solely the hepatic manifestation of the metabolic syndrome but arises as a result of network-wide perturbations in flux distributions at the organism-level, ultimately resulting in hepatic steatosis. The critical dependence of hepatic triglyceride concentration on inter-tissue transport reactions highlights the multi-compartmental nature of NAFLD and thus, disruption in the homeostatic balance of lipid distribution is at least partially causal of the disease state. The accumulation of hepatic triglycerides may initially be triggered due to a deregulation in pathway branch-points that are “intolerant” to flux alterations. The enrichment of these focal points in the adipose compartment for maintaining a balance in triglyceride synthesis, storage and hydrolysis, may indicate the initial role of adipose malfunction in triggering hepatic steatosis. The “threshold effect” displayed by these branch-points may be specific to individuals and may potentially explain the variability in NAFLD pathogenesis. This trigger may elicit functional changes in regulatory factors, such as FXR, which in turn activate a cascade of alterations in the rest of the network. In addition to confirming hepatic triglyceride sensitivity to previously identified deregulations in NAFLD, the SteatoNet identified novel candidate mechanisms, such as cholesterol transport, ketone body metabolism and regulatory functions of FXR, LXR and SREBP-2, that require experimental focus in the future. However, potential mechanisms that result in altered flux distributions remain an open question. Regulation of metabolic flux is a complex phenomenon that cannot be pinpointed to a single mechanism, as metabolic pathways are intricately connected and involve multiple enzyme isoforms with different substrates that are regulated by numerous factors at the transcriptional and translational level. Moreover, the inconsistency between the high prevalence of NAFLD and the small number of genetic variants identified in association with the disease indicates a critical role of environmental and dietary factors. The ability of SteatoNet to simulate the effect of the diet on metabolic homeostasis is a vital feature to investigate complex disorders and provides an advantage over currently available models. Dietary factors cause diverse changes in the organisms' metabolic status by triggering the activation or inhibition of regulators, such as hormones, cytokines and nuclear receptors [54]. SteatoNet analysis, which was conducted on a high-glucose and high-triglyceride intake background, highlighted the shift in the metabolic steady state on excessive dietary lipid or carbohydrate intake towards overproduction of the resulting metabolite substrates, which eventually causes enzyme saturation and a shift of metabolic flux into alternative pathways. Thus, individuals with genetic variations or inborn errors in metabolism affecting the activity or concentration of multiple enzymes or regulatory factors may be predisposed to hepatic steatosis as a result of inherent deregulations in flux distributions, with further adverse effects in the absence of suitable dietary measures.

In summary, we highlight the novelty of SteatoNet as a highly regulated multi-tissue platform, with the flexibility to systematically investigate genetic and dietary effects influencing hepatic metabolic homeostasis, in the absence of constrained kinetic parameters. SteatoNet computationally suggests that a single ‘hit’ is not sufficient to trigger hepatic steatosis. The concerted and cumulative action of various focal points in a systemic network are responsible to elicit significant phenotypic changes. SteatoNet thus promotes the application of basic engineering modelling strategies to solve complex biological questions in a simple and efficient manner.

## Methods

### Model generation

An object-oriented modelling and simulation programme based on the Modelica language, Dymola (Version 7.4, Dassault Systems, Lund, Sweden) [55], was utilised for the construction of SteatoNet in a systematic workflow summarised in Fig. 1. Dymola is best known in engineering industries but less in systems biology applications. An advantage of object-oriented modelling is the reusability of general equations and model object classes with a user-friendly graphical interface. We chose Dymola for the modelling purpose due to its ability to define user-specific model objects and libraries, handle large, dynamic, multi-domain models and generate simulations rapidly. A systems biology library of objects was generated based on differential algebraic equations (DAEs) corresponding to biological pathway entities, as described here and previously [3]. The basic objects of the library include enzymes, metabolites, non-enzymatic regulatory proteins, mRNAs, genes, flux sources and positive and negative regulatory objects for gene expression and protein activity (S1 Figure). SteatoNet was generated by compilation of these objects by linking them with connectors, thus forming a closed multi-pathway network (Fig. 2). The pathways in SteatoNet are based on their curation in Kyoto Encyclopaedia of Genes and Genomes (KEGG, http://www.genome.jp/kegg/) and the Reactome (www.reactome.org) databases. Regulation at the transcriptional and post-translational levels has also been incorporated following expert-based manual inspection of the literature. The literature was manually scanned (>500 articles) to select studies that identify and confirm regulatory interactions in various metabolic pathways prior to the incorporation of regulation in the SteatoNet. Putative interactions that have not been confirmed were not included in the model. The inclusion of nuclear receptors in the SteatoNet was based on their confirmed role in the glucose, lipid and amino acid metabolic pathways portrayed in the SteatoNet. The included nuclear receptors were verified for their regulatory role and ligands by extensive manual search of the literature and databases such as NURSA (Nuclear Receptor Signalling Atlas). Hence, only receptors with known expression, endogenous ligands and specific targets in either the liver/adipose/muscle tissues were included in the SteatoNet. Regulatory interactions that were unintentionally omitted were identified during the model validation procedure and incorporated in the model. Thus, the incorporation of the regulatory layer in the model was done in an iterative manner to ensure correspondence with biological behaviour.

The pathways included are glycolysis, gluconeogenesis, citric acid cycle, pentose phosphate pathway, *de novo* lipogenesis, β-oxidation, lipolysis, amino acid metabolism, ketone body synthesis and the transport of metabolites between the liver, adipose tissue, pancreas, other extra-hepatic tissues and macrophages *via* the blood. The external nutrient sources (influx of glucose, fats/triglycerides, cholesterol and essential amino acids) have been incorporated into the SteatoNet. These influxes represent the dietary intake and intestinal absorption of metabolites into the portal vein or secretion into the intestinal lymph (in the case of chylomicrons), which supply nutrients to the liver. The enzyme levels are governed by gene expression objects, which are regulated by transcription factors, such as PPARα, PPARγ, SREBP-1c, SREBP2, LXR, FXR, glucocorticoid receptor and PPARγ coactivators 1 alpha (PGC1A). The regulatory action of the hormones insulin and glucagon, the adipokines leptin and adiponectin and the cytokine TNFα has also been incorporated. The metabolites, enzymes and non-enzymatic regulatory proteins included in SteatoNet and the pathways they are associated with, are listed in S3, S4, S5 Tables S6 and S7 Tables provide detailed lists on the transcriptional and post-translational regulators of metabolism included in the model, their targets and the type of regulatory function elicited by them.

An open-access version of the SteatoNet along with the systems biology library has been included in the supplementary materials (S1 and S2 Datasets). These files can be accessed by the OpenModelica Connection Editor (OMEdit) software [56], which can be downloaded for free online (https://openmodelica.org/openmodelicaworld/tools). S1 Text provides instructions to access the model in OMEdit.

### Assignment of metabolic flux distributions

The distribution of fluxes at pathway branch points is defined by additional model equations that set the initial ratio of flux distribution from the parent pathway into each branch. Thus, the distribution of the metabolic flux, *f*, in each of the pathway branches is an additional independent parameter that must be specified in the model. Vo *et al* [57] generated a comprehensive flux network based on isotopomer tracer analysis in HepG2 cells. The estimated reaction fluxes from this study were utilised to approximate *f* at various SteatoNet branching points. The flux distribution proportion was calculated by summing the total flux at the branch-point and determining the proportion entering each branch as a fraction of the total flux. The choice of tracers in [57] and utilization of cell lines prevented identification of flux distributions in the lipid metabolism pathway, the transport of metabolites and distributions among tissues. Thus, at branch points with uncertainty in the value of *f*, an arbitrary value was assigned that resulted in stable model simulations. For several low tolerance branch-points, the value of *f* was not completely arbitrary as these focal points can tolerate only a low range of flux distributions.

The flux estimates from the Vo *et al* study were utilized to approximate *f* at branch-points within SteatoNet in order to gauge a physiological value for parameter assignment, while taking into consideration that *in vitro* isotopomer studies are subject to large variances due to differing cell culture conditions, sampling, pre-analytical processing, etc. [58]. It must be highlighted that due to the qualitative nature of the model and the normalization of model parameters, the assignment of values to the parameter *f* is critical in terms of model identification rather than quantitative reproduction of hepatic function. Hence, the assignment of arbitrary flux distribution values is feasible, provided that the model generates stable simulations and can simulate biological phenomena for validation purposes. In addition, immortal cell lines such as HepG2 display alterations in dynamics compared to primary cells and do not realistically depict organism-level flux distributions [59]. Consequently, the approximation of network flux distributions provides a reference stable steady state for comparison with model responses on perturbation, instead of precise quantitative estimations.

### Dynamic modelling and steady state analysis

The modelling method based on DAEs and analysis in steady-state utilised to build the model have been described previously [3]. The reaction dynamics (Fig. 3) is described by four equations in an extension to the Michaelis-Menten model of enzyme kinetics:
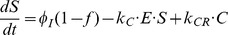
(1)

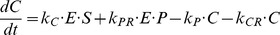
(2)

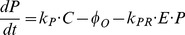
(3)

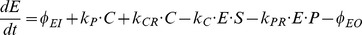
(4)
*S*, *E*, *C* and *P* denote the concentrations of the substrate, enzyme, enzyme-substrate (ES) complex and product, respectively. Constants *k_C_*, *k_P_*, *k_CR_* and *k_PR_* represent the rate constants of complex formation, product formation, ES complex dissociation into the enzyme and substrate and product reversion to the enzyme-product (EP) complex. *φ_I_*, *φ_O_*, *φ_EI_* and *φ_EO_* correspond to the substrate influx, the product efflux, the enzyme influx and the enzyme degradation flux. *f* denotes the proportion of the total substrate influx into alternative pathways.

Concentrations of *S*, *E*, *C* and *P* at steady-state are represented by *S_SS_*, *E_SS_*, *C_SS_* and *P_SS_*. These variables can be converted into dimensionless quantities by normalising them with their steady-state counterparts. Thus,
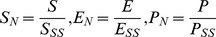
(5)The variables have been deliberately normalized to non-dimensional variables with the goal to observe relative changes of the variables. The normalization only affects the variable values while the systems dynamics remained unchanged. Extending the derivations described by Belič *et al* [3], an additional steady-state ratio is described to define the relative concentration of bound and free enzyme:
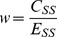
(6)The normalised value of the complex is determined in terms of the free enzyme concentration at steady state rather than the steady-state complex concentration. Thus,
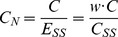
(7)Similar to derivations in [3], the steady state normalised values *S_N_*, *E_N_* and *P_N_* are equal to 1 and according to the relation described above, *C_N_ = w*. The rate constants are now described with incorporation of *w*:
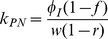
(8)

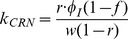
(9)

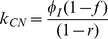
(10)

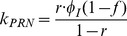
(11)All model variables can be uniquely calculated with the knowledge of the reversibility of the reaction *r*, the distribution of the influx *f* into alternative pathways, the total influx *φ_I_* and the ratio between the bound and free enzyme, *w*. To derive the relations of the variables at the new steady-state, 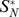, 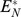, 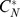, 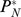 and f^*^ at which the system settles in an event of disturbance, we substitute these variables into the steady-state form of equations 1 and 2:
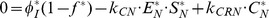
(12)

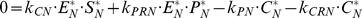
(13)This allows solving for the new steady-state concentration of the enzyme, substrate and product:
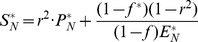
(14)

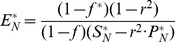
(15)

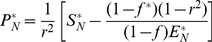
(16)
Equations 14–16 illustrate that apart from the classical interdependence between substrate, product and enzyme concentration, as stated by the Michaelis-Menten relations, the relative concentrations in the new steady-state depend also on the distribution of the influx into alternative pathway branches, but they do not directly depend on the absolute value of the total metabolic flux. The reversibility of a reaction, *r*, is an inherent property of the enzyme and the equilibrium constant between the species involved in the reaction. *r* represents the initial ratio of the reverse and forward metabolic flux, required to estimate the model parameters at the initial steady state. The parameter *r* was fixed based on literature searches for reactions with known reversibility ratios. For reactions with known reversibility but lack of specific defined ratios, *r* was fixed as 0.5 and for irreversible reactions *r* was fixed as 0. When the network is disturbed, a new steady state is reached where the reverse-forward flux ratio (when r>0 i.e. for reversible reactions) depends on the entire network dynamic properties and the type and magnitude of the disturbance. Most reactions in higher organisms have low reversibility, thus *r* is small, however, our simulation studies show that model performs well even for values of *r* above 0.5. Although, the squared form of *r* diminishes its influence on the concentration of the substrate, enzyme or product, it accounts for the thermodynamic constraints related to Gibb's free energy in the reactions. The flux distribution, f^*^, in metabolic pathways in the event of a disturbance adapts depending on the newly attained values of substrate influx, demand of downstream pathways and alterations in enzyme concentration in the acquired steady state and also on the initial network flux distribution. Thus, the value of f^*^ is calculated from the variable values at the new steady state attained after the disturbance.

### Gene expression regulation

In addition to the biological entities involved directly in metabolic reactions, feedback regulation was also incorporated into SteatoNet. mRNA transcription was modelled as a sigmoid function with separate object classes specified for positive and negative expression regulation. Negative expression control is described by the following equation:
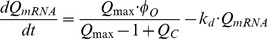
(17)where *Q_mRNA_* represents relative concentration of mRNA, *Q_max_* is the maximum relative mRNA expression, ***φ_O_*** is the transcription flux, *Q_C_* represents the concentration of the regulatory molecule and *k_d_* depicts the rate of mRNA degradation. Similarly, positive expression control is described by the following equation:
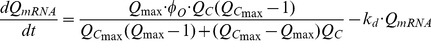
(18)where 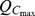 represents the maximum concentration of the regulator that results in the maximum fold-change in mRNA expression (*Q_max_*). The generation of protein is described as a linear relation between the relative concentration of mRNA and protein/enzyme quantity (*Q_P_*):
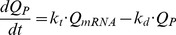
(19)where *k_t_* represents the rate constant of mRNA translation and *k_d_* is the rate constant of protein degradation. The quantity of the regulatory molecule (*Q_C_*) is usually controlled by activation or deactivation of the molecule by some metabolite quantity (*Q_M_*), which is described in a linear manner. The rate equation for protein activation is:
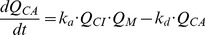
(20)The rate equation for protein inactivation is:
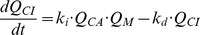
(21)Where *Q_CI_* and *Q_CA_* represent the pool of inactive and active protein, *k_i_* and *k_a_* are factors describing the activation or inhibition of the protein and the *k_d_* term in equations 20 and 21 accounts for the rate of protein degradation. Although the linear representation of translation and post-translational protein regulation provides a simplified depiction of the actual process, it sufficiently represents biological regulatory mechanisms and is considerably more informative compared to models without any feedback control.

### Model validation

To determine if the SteatoNet correctly depicts biological phenomena, simulations were compared to experimental data from the literature. The model was translated by the Microsoft visual studio 10.0 C compiler and simulations were generated by the default multi-step dassl solver in Dymola with a tolerance set to 1e-009. Model simulation is a multi-step numerical procedure that runs from time = 0 until arbitrary selected end time. In each step every equation of the model is evaluated. First, the derivatives of the model variables are calculated and next, the derivatives are used to change the values of the variables. The size of the simulation step is determined such that the derivatives of the variables are not too large since this would affect the simulation precision. In the case of the SteatoNet simulations, the dassl algorithm [60] was used. The selection of end time is crucial to observe the convergence to the new steady state and hence, must be selected such that all the model variables settle at a continual value, which is identified by observing the variables' time profiles.

Taking into consideration the assumptions of the model, the initial steady state concentration of all metabolites and enzymes is 1.0. On perturbation, the model variables are estimated based on the input values of *r*, *f*, *w* and ***φ_I_***. The simulations depict relative changes in the network components in response to triggers causing a shift from the initial steady state. The large number of model parameters in the SteatoNet presents an additional facet for model validation. In order to obtain further credibility of the model simulations, a parameter sensitivity analysis was performed to observe the sensitivity of simulation results with respect to *f*, as detailed in section 1.2.1. The effect of *r* has been previously tested on smaller models showing a minimal effect on model variables than *f*. *w* only affects the dynamics of transition from one steady-state to another and hence, does not influence the variable values at a particular steady-state. For the various substrate influxes in the SteatoNet (normalized as 1 at the initial steady state), several values of ***φ_I_*** have been tested and these directly affect variable values, as observed in the fasting (Fig. 4a) and high-fat diet simulations (Fig. 5). Thus, the values of *f* and ***φ_I_*** display the most prominent effect on model variable values in response to a disturbance.

### Sensitivity analysis

The dependence of model behaviour on parameters can be determined by sensitivity analysis, which is defined as the change in the model property versus the change in a parameter value. Metabolic control analysis (MCA) is an extension of local sensitivity analysis to determine the extent of change in metabolic flux or other systemic properties achieved by a fractional change in enzyme activity. MCA is quantified by control coefficients, which is termed as the flux control coefficient if the change in flux is considered as the model variable, or as the concentration control coefficient if the change in concentration is considered as the model variable. Relevant to this article and NAFLD-associated triglyceride accumulation, we can define the concentration control coefficient as the partial derivative of the change in triglyceride metabolite concentration with respect to small changes in the distribution of fluxes at various pathway branch-points. Thus, the sensitivity/concentration control coefficient is calculated by:
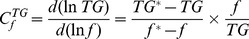
where 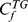 is the concentration control coefficient of parameter *f* with respect to hepatic triglyceride concentration. *TG* and TG* are the corresponding triglyceride concentrations at flux distribution values of *f* and f*. The second term in the equation i.e. the ratio between the initial flux distribution and triglyceride concentration, is incorporated to obtain relative sensitivity coefficients that are dimensionless. Graphically, the control coefficient is the tangent to the curve describing the relation between the metabolite concentration and model parameter variation and thus is dependent on the steady state under investigation. To determine the sensitivity of hepatic triglyceride concentration to the metabolic flux distribution, the glucose and triglyceride influx into the network was increased by 10-fold to simulate disturbance of the system on a high calorie diet. The distribution parameter for each branch point in the pathway model was varied by an interval of 10%. The corresponding changes in hepatic triglyceride synthesis were recorded and concentration control coefficients were calculated using equation 22.

## Supporting Information

S1 Figure Schematic representation of object classes in SysBio library. a) Metabolite substrate source class, b) metabolite class, c) enzyme class, d) non-enzymatic regulatory protein class, e) mRNA class, f) mRNA translation class, g) enzyme-catalysed reaction class, h) post-translational protein activation class, i) post-translational protein inhibition class, j) transcriptional activation class, k) transcriptional inhibition class. A-active protein port, C- Transcriptional regulator port, E-enzyme port, I (in f)-input port, I (in h and i)- Inactive protein port, O-output port, P-product port, S- substrate port.
(EPS)

S1 TablePathway branch-points with moderate 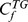 and low flux range tolerance.(DOCX)

S2 TablePathway branch-points with low 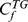 and low flux range tolerance.(DOCX)

S3 Table List of metabolites in SteatoNet and the metabolic pathways they are associated with.
(DOCX)

S4 Table List of enzymes in SteatoNet and the metabolic pathways they are associated with.
(DOCX)

S5 Table List of regulatory proteins in SteatoNet and the metabolic pathways they are associated with.
(DOCX)

S6 Table List of transcriptional regulators in SteatoNet, their target genes and the type of regulatory interaction.
(DOCX)

S7 Table List of post-translational regulators in SteatoNet, their target proteins and the type of regulatory interaction.
(DOCX)

S1 Dataset SteatoNet model file.
(RAR)

S2 Dataset Systems Biology library utilized to construct the SteatoNet.
(MO) Click here for additional data file.

S1 Text Instructions for accessing SteatoNet on Open Modelica Connnection Editor OMEdit.
(DOCX)
