# Supplementary material for: SteatoNet: The First Integrated Human Metabolic Model with Multi-layered Regulation to Investigate Liver-Associated Pathologies
Source: PLoS Comput Biol. 2014 Dec 11;10(12):e1003993. doi: 10.1371/journal.pcbi.1003993 (PMC4263370; doi:10.1371/journal.pcbi.1003993)
Supplement: S1 Table — Pathway branch-points with moderate and low flux range tolerance. (DOCX) [file pcbi.1003993.s002.docx]

**Table S1. Pathway branch-points with moderate** $\boldsymbol{C}_{\boldsymbol{f}}^{\boldsymbol{TG}}$ **and low flux range tolerance.**

| PATHWAY BRANCH | FLUX RANGE | SENSITIVITY RANGE |
| --- | --- | --- |
| G-6-P to G-1-P | Upto 40% of total flux into glucose | -0.6 to -0.2 |
| Cholesterol + SFA CoA to Cholesterol esters | Upto 10% of total flux into saturated fatty acyl CoA | -0.2 |
| FA_A_ (+ Gly-3-P) to LPA_A_ | Upto 30% of total flux into fatty acyl CoA_A_ | -0.5 to -0.14 |
| FA_B_ to MUFA_L_ | Upto 30% of total flux into serum fatty acids | 0.14 to 0.36 |
| TG_L_ to DAG_L_ | Upto 40% of total flux into liver triglycerides | 0.3 |

DAG- Diacylglycerol, FA- Fatty acids, G-6-P- Glucose-6-phosphate, G-1-P- Glucose-1-phosphate, Gly-3-P- Glycerol-3-phosphate, LPA- Lysophosphatidic acid, MUFA- Monounsaturated fatty acids, SFA- Saturated fatty acids, TG- Triglycerides, _A_- adipose, _B_- blood/serum, _L_-liver.
