# Supplementary material for: SteatoNet: The First Integrated Human Metabolic Model with Multi-layered Regulation to Investigate Liver-Associated Pathologies
Source: PLoS Comput Biol. 2014 Dec 11;10(12):e1003993. doi: 10.1371/journal.pcbi.1003993 (PMC4263370; doi:10.1371/journal.pcbi.1003993)
Supplement: S2 Table — Pathway branch-points with low and low flux range tolerance. (DOCX) [file pcbi.1003993.s003.docx]

## Table S2. Pathway branch-points with low $\boldsymbol{C}_{\boldsymbol{f}}^{\boldsymbol{TG}}$ and low flux range tolerance.

| PATHWAY BRANCH | FLUX RANGE | SENSITIVITY RANGE |
| --- | --- | --- |
| G-6-P to Ribulose-5-P | Upto 30% of total flux into glucose | -0.08 to -0.05 |
| Serine to Glycine | Upto 30% of total flux into serine | -0.00015 to 0.00056 |
| Serine+Homocysteine to Cystathionine | Upto 30% of total flux into serine | 0.00235 to 0.00475 |
| Glutamate to Glutamic semialdehyde | Upto 20% of total flux into glutamate | 0.0032 |
| DAG+Choline to Phosphatidylcholine | Upto 40% of total flux in DAG | 0.015 to 0.031 |
| Cholesterol + USFA CoA to Cholesterol esters | Upto10% of total flux into USFA CoA | -0.02 |
| Cholesterol_A_ synthesis | Upto 20% of total flux into acetyl CoA adipocyte | 0.00014 |
| Acetyl CoA_T_ to Malonyl CoA_T_ | Upto 30% of total flux into acetyl CoA tissue | -5.12E-06 to -7.89E-06 |
| Cholesterol_T_ synthesis | Upto 20% of total flux into acetyl CoA tissue | 0.000102 |
| DHAP to Gly-3-P | Upto 20% of total flux into DHAP | -0.07 |
| DAG to MAG | Upto 30% of total flux into DAG | -0.1 to -0.04 |

DAG- Diacylglycerol, DHAP- Dihydroxyacetone phosphate, FA- Fatty acids, G-6-P- Glucose-6-phosphate, G-1-P- Glucose-1-phosphate, Gly-3-P- Glycerol-3-phosphate, LPA- Lysophosphatidic acid, MAG- Monoacylglycerol, MUFA- Monounsaturated fatty acids, SFA- Saturated fatty acids, TG- Triglycerides, _A_- adipose, _B_- blood/serum, _L_-liver.
