# Supplementary material for: SteatoNet: The First Integrated Human Metabolic Model with Multi-layered Regulation to Investigate Liver-Associated Pathologies
Source: PLoS Comput Biol. 2014 Dec 11;10(12):e1003993. doi: 10.1371/journal.pcbi.1003993 (PMC4263370; doi:10.1371/journal.pcbi.1003993)
Supplement: S3 Table — List of metabolites in SteatoNet and the metabolic pathways they are associated with. (DOCX) [file pcbi.1003993.s004.docx]

**Table S3. List of metabolites in SteatoNet and the metabolic pathways they are associated with.**

| **METABOLITE** | **PATHWAY** |
| --- | --- |
| Glucose-1-phosphate | Gluconeogenesis/Glucose metabolism |
| Glucose-6-phosphate | Glycolysis/Glucose metabolism |
| Fructose-6-phosphate | Glycolysis/Glucose metabolism |
| Fructose-1,6-bisphospahte | Glycolysis/Glucose metabolism |
| Glyceraldehyde-3-phosphate | Glycolysis/Glucose metabolism |
| Bis-1,3-phosphoglycerate | Glycolysis/Glucose metabolism |
| Glycerate-3-phospahte | Glycolysis/Glucose metabolism |
| Glycerate-2-phosphate | Glycolysis/Glucose metabolism |
| Phosphoenol pyruvate | Glycolysis/Glucose metabolism |
| Pyruvate | Glycolysis/Glucose metabolism |
| Acetyl CoA (mitochondrial) | Citric acid cycle /Glucose metabolism |
| Oxaloacetate | Citric acid cycle /Gluconeogenesis/Glucose metabolism |
| Palmitoleate | Fatty acid metabolism |
| Acylcarnitine | β-oxidation/ Fatty acid metabolism |
| Trans2BEnoyl CoA | β-oxidation/ Fatty acid metabolism |
| L3Hydroxyacyl CoA | β-oxidation/ Fatty acid metabolism |
| Keto3acylCoA | β-oxidation/ Fatty acid metabolism |
| UDP-Glucose | Gluconeogenesis/Glucose metabolism |
| Glycogen | Gluconeogenesis/Glucose metabolism |
| Malonyl CoA | *de novo* lipogenesis /Glucose metabolism |
| Citrate | Citric acid cycle/Glucose metabolism |
| Acetyl CoA (cytoplasm) | /Glucose metabolism |
| Palmitate | Fatty acid metabolism |
| Palmitoleoyl CoA | Fatty acid metabolism |
| Lysophosphatidic Acid | Lipogenesis/Fatty acid metabolism |
| Glycerol-3-phosphate | Lipogenesis/Fatty acid metabolism/Glucose metabolism |
| Phosphatidic Acid | Lipogenesis/Fatty acid metabolism |
| Diacylglycerol | Lipogenesis/Fatty acid metabolism |
| Triglycerides | Lipogenesis/Fatty acid metabolism |
| Dihydroxyacetone phosphate | Glycolysis/ Glucose metabolism |
| Cholesterol | Cholesterol metabolism |
| Lactate | Glucose metabolism |
| Blood glucose | Glucose metabolism |
| Hepatic Glucose | Glucose metabolism |
| Oxalosuccinate | Citric acid cycle /Glucose metabolism |
| Isocitrate | Citric acid cycle /Glucose metabolism |
| CisAconitate | Citric acid cycle /Glucose metabolism |
| Oxoglutarate | Citric acid cycle /Glucose metabolism |
| S-Succinyl dihydrolipamide E | Citric acid cycle /Glucose metabolism |
| Succinyl CoA | Citric acid cycle /Glucose metabolism |
| Succinate | Citric acid cycle /Glucose metabolism |
| Fumarate | Citric acid cycle /Glucose metabolism |
| Malate (mitochondrial) | Citric acid cycle /Glucose metabolism |
| VLDL | Lipogenesis/Fatty acid metabolism |
| Alanine | Amino acid metabolism |
| Aspartate | Amino acid metabolism |
| Blood Fatty acid | Fatty acid metabolism |
| Chylomicron Triglycerides | Fatty acid metabolism |
| Monoacylglycerol | Fatty acid metabolism |
| Glycerol | Lipogenesis/Fatty acid metabolism/Glucose metabolism |
| Mito FattyAcyl CoA | β-oxidation/ Fatty acid metabolism |
| Acetoacetyl CoA | Ketone body metabolism |
| HMG CoA | Ketone body metabolism |
| Hepatic Acetoacetate | Ketone body metabolism |
| Heptic BHydroxybutyrate | Ketone body metabolism |
| Blood BHydroxybutyrate | Ketone body metabolism |
| Blood Acetoacetate | Ketone body metabolism |
| Tissue BHydroxybutyrate | Ketone body metabolism |
| Tissue Acetoacetate | Ketone body metabolism |
| Tissue Acetoacetyl CoA | Ketone body metabolism |
| Tissue Acetyl CoA | Glucose metabolism |
| Adipocyte BHydroxybutyrate | Ketone body metabolism |
| Adipocyte Acetoacetate | Ketone body metabolism |
| Adipocyte Acetoacetyl CoA | Ketone body metabolism |
| Adipocyte Acetyl CoA | Glucose metabolism |
| Adipocyte fatty acids | Lipogenesis/Fatty acid metabolism |
| Adipocyte Unsaturated Fatty acyl CoA | Lipogenesis/Fatty acid metabolism |
| Adipocyte Glycerol-3-phosphate | Lipogenesis/Fatty acid metabolism |
| Adipocyte Lysophosphatidic Acid | Lipogenesis/Fatty acid metabolism |
| Adipocyte Phosphatidic Acid | Lipogenesis/Fatty acid metabolism |
| Adipocyte diacylglycerol | Lipogenesis/Fatty acid metabolism |
| Adipocyte Triglycerides | Lipogenesis/Fatty acid metabolism |
| Adipocyte Glycerol | Lipogenesis/Fatty acid metabolism/Glucose metabolism |
| Adipocyte Monoacylglycerol | Lipogenesis/Fatty acid metabolism |
| Tissue Fatty acid | Fatty acid metabolism |
| Fructose-2,6-Bisphosphate | Glucose metabolism |
| Adipocyte Glucose | Glucose metabolism |
| Adipocyte glucose-6-phosphate | Glucose metabolism |
| Adipocyte Fructose-6-phosphate | Glucose metabolism |
| Adipocyte Fructose-1,6-Bisphosphate | Glucose metabolism |
| Adipocyte Dihydroxyacetone phosphate | Glucose metabolism |
| Adipocyte Glyceraldehyde-3-phosphate | Glucose metabolism |
| Adipocyte Pyruvate | Glucose metabolism |
| Tissue Glucose | Glucose metabolism |
| Tissue glucose-6-phosphate | Glucose metabolism |
| Tissue Fructose-6-phosphate | Glucose metabolism |
| Tissue Fructose-1,6-Bisphosphate | Glucose metabolism |
| Tissue Dihydroxyacetone phosphate | Glucose metabolism |
| Tissue Glyceraldehyde-3-phosphate | Glucose metabolism |
| Tissue Pyruvate | Glucose metabolism |
| HMG CoA (cytoplasmic) | Glucose metabolism/ Cholesterol metabolism |
| Acetoacetyl CoA (cytoplasmic) | Cholesterol metabolism |
| Blood Cholesterol | Cholesterol metabolism |
| Blood LDL-cholesterol | Cholesterol metabolism |
| Adipocyte Cholesterol | Cholesterol metabolism |
| Tissue Cholesterol | Cholesterol metabolism |
| Macrophage Cholesterol | Cholesterol metabolism |
| Pre-HDL | Lipoprotein metabolism |
| A4-HDL | Lipoprotein metabolism |
| A2-HDL | Lipoprotein metabolism |
| A3-HDL | Lipoprotein metabolism |
| Cholesterol ester-rich VLDL | Lipoprotein metabolism |
| Triglyceride-rich A1-HDL | Lipoprotein metabolism |
| Blood Cholesterol (from peripheral tissues) | Lipoprotein metabolism |
| Adipocyte Malonyl CoA | Fatty acid metabolism |
| tissue Malonyl CoA | Fatty acid metabolism |
| Ribulose-5-phosphate | Pentose phosphate pathway |
| Ribose-5-phosphate | Pentose phosphate pathway |
| Xylulose-5-phosphate | Pentose phosphate pathway |
| Sedoheptulose-7-phosphate | Pentose phosphate pathway |
| Erythrose-4-phosphate | Pentose phosphate pathway |
| Glutamate | Amino acid metabolism |
| Glutamine | Amino acid metabolism |
| Asparagine | Amino acid metabolism |
| Glutamate-5-phosphate | Amino acid metabolism |
| Glutamic Semialdehyde | Amino acid metabolism |
| Proline | Amino acid metabolism |
| Arginine | Amino acid metabolism |
| Phospho-3-hydroxy pyruvate | Amino acid metabolism |
| Phospho-3-serine | Amino acid metabolism |
| Serine | Amino acid metabolism |
| Glycine | Amino acid metabolism |
| Methionine | Amino acid metabolism |
| Homocysteine | Amino acid metabolism |
| Cystathionine | Amino acid metabolism |
| Cysteine | Amino acid metabolism |
| α-ketobutyrate | Amino acid metabolism |
| Phenylalanine | Amino acid metabolism |
| Tyrosine | Amino acid metabolism |
| Ammonia | Ammonia cycle |
| Valine | Amino acid metabolism |
| Propionyl CoA | Amino acid metabolism |
| Methylmalonyl CoA | Amino acid metabolism |
| Isoleucine | Amino acid metabolism |
| Histidine | Amino acid metabolism |
| Leucine | Amino acid metabolism |
| Tryptophan | Amino acid metabolism |
| Hydroxy-3-Anthranilate | Amino acid metabolism |
| Carbamoyl phosphate | Amino acid metabolism |
| Ornithine | Amino acid metabolism |
| Citrulline | Amino acid metabolism |
| Argininosuccinate | Amino acid metabolism |
| Urea | Urea cycle |
| Acetate | Glucose metabolism |
| Acetone | Ketone body metabolism |
| Phosphatidyl choline | Glycerophospholipid metabolism |
| Choline | Glycerophospholipid metabolism |
| Keto-3-Sphingosine | Sphingolipid metabolism |
| Sphinganine | Sphingolipid metabolism |
| Dihydroceramide | Fatty acid metabolism |
| Ceramide | Sphingolipid metabolism |
| Palmitoyl CoA | Lipogenesis/Fatty acid metabolism |
| Cholesterol esters | Cholesterol metabolism/Lipogenesis |
| Steroids | Cholesterol metabolism |
| Bile Acids | Cholesterol metabolism |
| Adipocyte Saturated Fatty Acyl CoA | Lipogenesis/Fatty acid metabolism |
| Adipocyte Triglycerides (lipid droplets) | Lipogenesis/Fatty acid metabolism |
| Chylomicron | Lipogenesis/Fatty acid metabolism |
| Hepatic Triglycerides (lipid droplet) | Lipogenesis/Fatty acid metabolism |
