# Supplementary material for: SteatoNet: The First Integrated Human Metabolic Model with Multi-layered Regulation to Investigate Liver-Associated Pathologies
Source: PLoS Comput Biol. 2014 Dec 11;10(12):e1003993. doi: 10.1371/journal.pcbi.1003993 (PMC4263370; doi:10.1371/journal.pcbi.1003993)
Supplement: S4 Table — List of enzymes in SteatoNet and the metabolic pathways they are associated with. (DOCX) [file pcbi.1003993.s005.docx]

**Table S4. List of enzymes in SteatoNet and the metabolic pathways they are associated with.**

| **ENZYME** | **PATHWAY** |
| --- | --- |
| A 4 HDL formation enzyme | Lipoprotein metabolism |
| Adipocyte ABCA1 | Fatty acid transport |
| Tissue ABCA1 | Fatty acid transport |
| Acetoacetate decarboxylase | Ketone bodies metabolism |
| Acetoacetyl CoA thiolase + HMGCoA Synthase cyto | Cholesterol metabolism |
| Acetone elimination enzyme | Ketone bodies metabolism |
| Tissue Acetyl CoA carboxylase | De novo lipogenesis |
| Acetyl CoA synthase | Glucose metabolism |
| Aconitate Hydratase | Citric acid cycle |
| Acetyl CoA carboxylase | De novo lipogenesis |
| Adipose Acetyl CoA carboxylase | De novo lipogenesis |
| Pyruvate carboxylase | Citric acid cycle |
| Acyl CoA:Cholesterol Acyltransferase | Cholesterol metabolism |
| Acyl CoA Dehydrogenase | Fatty acid oxidation |
| Glycogen Synthase Kinase-3β | Glycogen metabolism |
| Alanine Aminotransferase | Alanine metabolism |
| Aldolase | Glycolysis |
| Adipose Aldolase | Glycolysis |
| Tissue Aldolase | Glycolysis |
| Alpha ketoacid dehydrogenase | Amino acid metabolism |
| Alpha ketobutyrate Elimination enzyme | Amino acid metabolism |
| ApolipoproteinA1 | Lipoprotein metabolism |
| Arginase | Arginine metabolism |
| Arginine synthesis enzymes | Arginine metabolism |
| Argininosuccinase | Arginine metabolism |
| Argininosuccinate synthetase | Arginine metabolism |
| SREBP cleavage activating protein | Cholesterol metabolism |
| Asparagine synthase | Asparagine metabolism |
| Aspartate Aminotransferase | Aspartate metabolism |
| ATP Citrate lyase | Citric acid cycle |
| Tissue β-oxidation enzymes | Fatty acid oxidation |
| Adipose β-oxidation enzymes | Fatty acid oxidation |
| Β-Ketothiolase | Fatty acid oxidation |
| Adipose β-Ketothiolase | Fatty acid oxidation |
| Tissue β-Ketothiolase | Fatty acid oxidation |
| Adipose β-Hydroxybutyrate + Acetoacetate transporter | Ketone bodies transport |
| Liver β-Hydroxybutyrate + Acetoacetate transporter to blood | Ketone bodies transport |
| Tissue β-Hydroxybutyrate + Acetoacetate transporter | Ketone bodies transport |
| β-Hydroxybutyrate Dehydrogenase | Ketone bodies metabolism |
| Tissue β-Hydroxybutyrate Dehydrogenase | Ketone bodies metabolism |
| Adipose β-Hydroxybutyrate Dehydrogenase | Ketone bodies metabolism |
| Bile acid synthesis enzymes | Cholesterol metabolism |
| Steroids synthesis enzymes | Cholesterol metabolism |
| Adipose β-KetoAcyl CoA Transferase | Ketone bodies metabolism |
| Tissue β-KetoAcyl CoA Transferase | Ketone bodies metabolism |
| Carbamoylphosphate synthetase | Ammonia metabolism |
| Carnitine Acyltransferase I | Fatty acid oxidation |
| Carnitine Acyltransferase II | Fatty acid oxidation |
| Liver Caveolin FATP CD36 FABP | Fatty acid transport |
| Tissue CD36 | Fatty acid transport |
| Choesteryl ester utilization enzymes | Cholesterol metabolism |
| Ceramide Utilization enzymes | Sphingolipid metabolism |
| Cholesterol ester transfer protein | Lipoprotein metabolism |
| Macrophage Cholesterol storage enzymes | Cholesterol metabolism |
| HMGCR | Cholesterol metabolism |
| Adipose Cholesterol synthesis enzymes | Cholesterol metabolism |
| Tissue Cholesterol synthesis enzymes | Cholesterol metabolism |
| Adipose Cholesterol utilization enzymes | Cholesterol metabolism |
| Intestine Cholesterol absorption enzymes | Cholesterol metabolism |
| Tissue Cholesterol utilization enzymes | Cholesterol metabolism |
| Liver Cholesterol utilization enzymes | Cholesterol metabolism |
| Choline-diacylglycerol choline phosphotransferase | Glycerophospholipid metabolism |
| Choline elimination enzyme | Glycerophospholipid metabolism |
| Chylomicron receptor liver | Lipoprotein metabolism |
| Citrate Synthase | Citric acid cycle |
| CYP51A1 | Cholesterol metabolism |
| Cystathionine synthase | Serine metabolism |
| Cystathionine lyase | Serine/Cysteine metabolism |
| DAG utilization enzyme | Triglyceride metabolism |
| DAG Acyltransferase | Triglyceride metabolism |
| Adipose DAG Acyltransferase | Triglyceride metabolism |
| DHCR-14 | Cholesterol metabolism |
| DHCR-24 | Cholesterol metabolism |
| DHCR-7 | Cholesterol metabolism |
| Squalene Synthase + Lanosterol Synthase | Cholesterol metabolism |
| SC4MOL, NSDHL1, HSD3B3, EBP | Cholesterol metabolism |
| Mevalonate Kinase, Phosphomevalonate kinase, Mevalonate-5-Pyrophosphate decarboxylase | Cholesterol metabolism |
| Enolase | Glycolysis |
| Enoyl CoA Hydratase, L3-Hydroxy acyl CoA Dehydrogenase | Fatty acid oxidation |
| Adipose CD36 transporter | Fatty acid transport |
| Fatty Acid Synthase | De novo lipogenesis |
| Adipose Fatty Acid Synthase | De novo lipogenesis |
| Tissue Fatty Acid Synthase | De novo lipogenesis |
| Fatty Acyl CoA Synthetase | Fatty acid metabolism |
| Adipose Fatty Acyl CoA Synthetase | Fatty acid metabolism |
| Fructose-1,6-Bisphosphatase | Gluconeogenesis |
| Fructose-2,6-Bisphosphatase | Glycolysis |
| Fumarate hydratase | Citric acid cycle |
| γ-Glutamyl Transferase, Glutamate-5-Phosphate dehydrogenase | Amino acid metabolism / Urea cycle |
| Glucokinase | Glycolysis |
| Glucokinase Regulatory Protein | Glycolysis |
| Adipose GLUT4 | Glucose transport |
| Liver GLUT1 | Glucose transport |
| Liver GLUT4 | Glucose transport |
| Glucose-6-Phosphate Dehydrogenase, Lactonase, 6-Phosphogluconate Dehydrogenase | Pentose Phosphate Pathway |
| Glucose-6-Phosphatase | Glycolysis |
| Tissue GLUT4 | Glucose transport |
| Glutamate Dehydrogenase | Amino acid metabolism |
| Glutamic Semialdehyde aminotransferase | Amino acid metabolism |
| Glutaminase | Amino acid metabolism |
| Glutamine synthetase | Amino acid metabolism |
| Glyceraldehyde-3-Phosphate Dehydrogenase, Phospho-glycerate kinase, Phospho-glycerate mutase | Glycolysis |
| Adipose Glyceraldehyde-3-Phosphate Dehydrogenase, Phospho-glycerate kinase, Phospho-glycerate mutase, Enolase, Pyruvate kinase | Glycolysis |
| Tissue Glyceraldehyde-3-Phosphate Dehydrogenase, Phospho-glycerate kinase, Phospho-glycerate mutase, Enolase, Pyruvate kinase | Glycolysis |
| Glycerokinase | Triglyceride metabolism |
| Adipose Glycerokinase | Triglyceride metabolism |
| Glycerol elimination enzyme | Triglyceride metabolism |
| Glycerol-3-Phosphate Acyltransferase | Triglyceride metabolism |
| Adipose Glycerol-3-Phosphate Acyltransferase | Triglyceride metabolism |
| Glycerol-3-Phosphate Dehydrogenase | Triglyceride metabolism |
| Adipose Glycerol-3-Phosphate Dehydrogenase | Triglyceride metabolism |
| Glycerophospholipid synthesis enzymes | Glycerophospholipid metabolism |
| Glycogen Phosphorylase | Glycogen metabolism |
| Glycogen Synthase | Glycogen metabolism |
| Hepatic lipase, Endothelial lipase | Triglyceride metabolism |
| Adipose Hexokinase, phosphoglucoisomerase, Phosphofructokinase | Glycolysis |
| Tissue Hexokinase, phosphoglucoisomerase, Phosphofructokinase | Glycolysis |
| Histidine degradation enzymes | Amino acid metabolism |
| HMG CoA Lyase | Ketone bodies metabolism |
| HMGCoA Synthase | Ketone bodies metabolism |
| Hormone sensitive Lipase | Triglyceride metabolism |
| Adipose Hormone Sensitive lipase | Triglyceride metabolism |
| Isocitrate Dehydrogenase | Citric acid cycle |
| Isoleucine to Propionyl CoA enzymes | Amino acid metabolism |
| Keto3Sphingosine Reductase, Ceramide Synthase, Dihydroceramide Desaturase | Sphingolipid metabolism |
| Lactate Dehydrogenase | Glucose metabolism |
| Lactate utilization enzyme | Glucose metabolism |
| Adipose LDL receptor | Lipoprotein metabolism |
| Liver LDL receptor | Lipoprotein metabolism |
| Macrophage LDL receptor, CD36 | Lipoprotein metabolism |
| Tissue LDL receptor | Lipoprotein metabolism |
| Lecithin cholesterol acyltransferase | Lipoprotein metabolism |
| Leucine utilization enzymes | Amino acid metabolism |
| Lipoprotein Lipase active and CD36 | Lipoprotein metabolism |
| Adipose Lysophosphatidic acid Acyltransferase (LPAT) | Triglyceride metabolism |
| Liver Lysophosphatidic acid Acyltransferase, AGPAT | Triglyceride metabolism |
| Adipose monoacylglycerol synthase | Triglyceride metabolism |
| Adipose Monoacylglycerol acyltransferase | Triglyceride metabolism |
| Malate Dehydrogenase | Citric acid cycle |
| Malic enzyme | Citric acid cycle |
| Malonyl CoA decarboxylase | Glucose metabolism |
| Methionine adenosyltransferase | Amino acid metabolism |
| Methionine synthase | Amino acid metabolism |
| Methylmalonyl CoA mutase | Amino acid metabolism |
| Liver Monoacylglycerol acyltransferase | Triglyceride metabolism |
| Adipose Monoglyceride lipase | Triglyceride metabolism |
| MTTP | Triglyceride metabolism |
| Ornithine transcarbamoylase | Amino acid metabolism |
| Ornithine utilization enzymes | Amino acid metabolism |
| Oxoglutarate-2-dehydrogenase E1 component | Citric acid cycle |
| Oxoglutarate-2-dehydrogenase E2 component | Citric acid cycle |
| Phosphatidic acid Phosphatase | Triglyceride metabolism |
| Adipose Phosphatidic acid Phosphatase | Triglyceride metabolism |
| Phosphatidylcholine breakdown enzyme | Glycerophospholipid metabolism |
| Phosphoenol pyruvate carboxykinase | Glycolysis |
| Phenylalanine hydroxylase | Amino acid metabolism |
| Phospho-6-fructokinase 2 | Gluconeogenesis |
| Phosphofructokinase | Glycolysis |
| Phosphoglucoisomerase | Glycolysis |
| Phosphoglucomutase | Glucose metabolism |
| Phosphoglycerate Dehydrogenase | Amino acid metabolism |
| Phosphopentose epimerase | Pentose Phosphate Pathway |
| Phosphopentose isomerase | Pentose Phosphate Pathway |
| Phosphorylase kinase | Glucose metabolism |
| Protein kinase A | Glucose metabolism |
| Adipose Protein kinase A | Glucose metabolism/ Triglyceride metabolism |
| Propionyl CoA carboxylase | Amino acid metabolism |
| Adipose Protein Phosphatase1 | Glucose metabolism/ Triglyceride metabolism |
| Glutamate utilization/degradation enzymes | Amino acid metabolism |
| Arginine utilization/degradation enzymes | Amino acid metabolism |
| Methionine utilization/degradation enzymes | Amino acid metabolism |
| Tyrosine utilization/degradation enzymes | Amino acid metabolism |
| Phenylalanine utilization/degradation enzymes | Amino acid metabolism |
| Isoleucine utilization/degradation enzymes | Amino acid metabolism |
| Valine utilization/degradation enzymes | Amino acid metabolism |
| Propionyl CoA utilization/degradation enzymes | Amino acid metabolism |
| Histidine utilization/degradation enzymes | Amino acid metabolism |
| Leucine utilization/degradation enzymes | Amino acid metabolism |
| Tryptophan utilization/degradation enzymes | Amino acid metabolism |
| Glutamine utilization/degradation enzymes | Amino acid metabolism |
| Proline utilization/degradation enzymes | Amino acid metabolism |
| Aspartate utilization/degradation enzymes | Amino acid metabolism |
| Alanine utilization/degradation enzymes | Amino acid metabolism |
| Asparagine utilization/degradation enzymes | Amino acid metabolism |
| Serine utilization/degradation enzymes | Amino acid metabolism |
| Glycine utilization/degradation enzymes | Amino acid metabolism |
| Cysteine utilization/degradation enzymes | Amino acid metabolism |
| Adipose Pyruvate dehydrogenase | Glycolysis |
| Tissue Pyruvate dehydrogenase | Glycolysis |
| Pyruvate ferredoxin oxidoreductase | Glycolysis |
| Pyruvate kinase | Glycolysis |
| Pyyroline-5-Carboxylate Reductase | Amino acid metabolism |
| SCRB1 receptor | Lipoprotein metabolism |
| Serine Aminotransferase | Amino acid metabolism |
| Serine Dehydratase | Amino acid metabolism |
| Serine palmitoyltransferase | Amino acid metabolism |
| Serine Phosphatase | Amino acid metabolism |
| Serine Transhydroxymethylase | Amino acid metabolism |
| Stearoyl CoA Desaturase | Fatty acid metabolism |
| Adipose Stearoyl CoA Desaturase | Fatty acid metabolism |
| Steroid utilization enzymes | Cholesterol metabolism |
| succinate dehydrogenase ubiquinone | Citric acid cycle |
| Succinyl CoA acetoacetate CoA transferase | Citric acid cycle |
| Succinyl CoA synthetase alpha subunit | Citric acid cycle |
| Adipose TCA cycle enzymes | Citric acid cycle |
| Tissue TCA cycle enzymes | Citric acid cycle |
| Liver Triglyceride (Lipid Droplets) release enzyme | Triglyceride metabolism |
| Liver Triglyceride storage (in Lipid Droplets) enzyme | Triglyceride metabolism |
| Liver Triglyceride utilization enzyme | Triglyceride metabolism |
| Transaldolase | Pentose phosphate pathway |
| Transketolase | Pentose phosphate pathway |
| Adipose Triglyceride storage enzymes | Triglyceride metabolism |
| Triose Phosphoisomerase | Glycolysis |
| Adipose Triose Phosphoisomerase | Glycolysis |
| Tissue Triose Phosphoisomerase | Glycolysis |
| Tryptophan to Hydroxy-3-Anthranilate enzymes (Kynureninase) | Amino acid metabolism |
| Hydroxy-3-Anthranilate to Acetoacetyl CoA enzyme (3-hydroxyanthranilic acid oxygenase, 2-amino-3-carboxymuconate-6-semialdehyde decarboxylase) | Amino acid metabolism |
| Tyrosine to acetoacetate and fumarate enzymes (Fumarylacetoacetase) | Amino acid metabolism |
| Ubiquitination complex | Post-translational modification |
| UDP-Glucose Pyrophosphorylase | Glycogen metabolism |
| Urea cycle enzymes | Urea cycle |
| Urea excretion enzymes | Urea cycle |
| Valine to Propionyl CoA enzymes | Amino acid metabolism |
