# Supplementary material for: SteatoNet: The First Integrated Human Metabolic Model with Multi-layered Regulation to Investigate Liver-Associated Pathologies
Source: PLoS Comput Biol. 2014 Dec 11;10(12):e1003993. doi: 10.1371/journal.pcbi.1003993 (PMC4263370; doi:10.1371/journal.pcbi.1003993)
Supplement: S5 Table — List of regulatory proteins in SteatoNet and the metabolic pathways they are associated with. (DOCX) [file pcbi.1003993.s006.docx]

**Table S5. List of regulatory proteins in SteatoNet and the metabolic pathways they are associated with.**

| **PROTEIN** | **PATHWAY** |
| --- | --- |
| Active and inactive hepatic AKT | Glucose metabolism, Insulin signalling |
| Active and inactive tissue AKT | Glucose metabolism, Insulin signalling |
| Active and inactive SREBP1c | *De novo* lipogenesis, Fatty acid metabolism |
| Active and inactive PPARA | Fatty acid metabolism |
| Active and inactive LXRA | Fatty acid metabolism |
| Blood insulin | Glucose metabolism, Insulin signalling |
| Blood glucagon | Glucose metabolism |
| Active and inactive SREBP2 | Cholesterol metabolism |
| Active and inactive hepatic insulin receptor substrates | Glucose metabolism, Insulin signalling |
| Active and inactive hepatic insulin receptor | Glucose metabolism, Insulin signalling |
| Active and inactive adipocyte insulin receptor | Glucose metabolism, Insulin signalling |
| Active and inactive tissue insulin receptor | Glucose metabolism, Insulin signalling |
| Adiponectin | Glucose metabolism, Fatty acid metabolism, Adipokine signalling |
| Active and inactive AdipoR1 | Glucose metabolism, Fatty acid metabolism, Adipokine signalling |
| Active and inactive AdipoR2 | Glucose metabolism, Fatty acid metabolism, Adipokine signalling |
| Active and inactive AMPK | Glucose metabolism, Fatty acid metabolism, Adipokine signalling |
| Active and inactive tissue AMPK | Glucose metabolism, Fatty acid metabolism, Adipokine signalling |
| Active and inactive FOXO1 | Insulin signalling |
| Active and inactive tissue FOXO1 | Insulin signalling |
| Active and inactive adipocyte TNF-α | Adipokine signalling |
| Active and inactive Leptin | Adipokine signalling |
| Active and inactive adipocyte mTOR | Insulin signalling, Adipokine signalling |
| Active and inactive liver TNF-α | Adipokine signalling |
| Active and inactive ChREBP | Glucose metabolism |
| Active and inactive PGC1A | Insulin signalling, Adipokine signalling |
| Active and inactive FXR | Bile acid metabolism |
| Active and inactive PPARγ | Fatty acid metabolism |
| Active and inactive macrophage TNF-α | Adipokine signalling |
| Active and inactive TLR4 | Fatty acid metabolism |
| Active and inactive adipocyte SREBP1c | *De novo* lipogenesis, Fatty acid metabolism |
| Active and inactive Glucocorticoid receptor | Glucose metabolism, Fatty acid metabolism |
