# Supplementary material for: SteatoNet: The First Integrated Human Metabolic Model with Multi-layered Regulation to Investigate Liver-Associated Pathologies
Source: PLoS Comput Biol. 2014 Dec 11;10(12):e1003993. doi: 10.1371/journal.pcbi.1003993 (PMC4263370; doi:10.1371/journal.pcbi.1003993)
Supplement: S6 Table — List of transcriptional regulators in SteatoNet, their target genes and the type of regulatory interaction. (DOCX) [file pcbi.1003993.s007.docx]

**Table S6. List of transcriptional regulators in SteatoNet, their target genes and the type of regulatory interaction.**

| **TRANSCRIPTIONAL TARGET** | **REGULATOR** | **TYPE OF REGULATION** |
| --- | --- | --- |
| Glucokinase | SREBP-1c | positive |
| Glucokinase | Fructose-2,6-BP | Positive |
| Glucose-6-phosphatase | Glucagon | Positive |
| Glucose-6-phosphatase | FOXO1 | Positive |
| Glucose-6-phosphatase | PGC1A | Positive |
| Glucose-6-phosphatase | Glucocorticoid | Positive |
| Glucose-6-phosphatase | Insulin | Negative |
| Glucose-6-phosphatase | AMPK | Negative |
| Fructose-1,6-bisphosphatase | Insulin | Negative |
| Fructose-1,6-bisphosphatase | PGC1A | Positive |
| PEPCK | Glucagon | Positive |
| PEPCK | FOXO1 | Positive |
| PEPCK | Glucocorticoid | Positive |
| PEPCK | Insulin | Negative |
| PEPCK | AMPK | Negative |
| Pyruvate kinase | Insulin | Positive |
| Pyruvate kinase | ChREBP | Positive |
| Pyruvate kinase | Glucagon | Negative |
| Fatty acid synthase | SREBP-1c | Positive |
| Fatty acid synthase | Insulin | Positive |
| Fatty acid synthase | LXR-α | Positive |
| Fatty acid synthase | ChREBP | Positive |
| Fatty acid synthase | PGC1A | Negative |
| Acetyl CoA carboxylase | SREBP-1c | Positive |
| Acetyl CoA carboxylase | Insulin | Positive |
| Acetyl CoA carboxylase | LXR-α | Positive |
| Acetyl CoA carboxylase | ChREBP | Positive |
| Acetyl CoA carboxylase | Glucocorticoid | Positive |
| Β-ketothiolase | PPARα | Positive |
| Β-ketothiolase | Glucagon | Positive |
| Enoyl CoA Hydratase | PPARα | Positive |
| Acyl CoA dehydrogenase | PPARα | Positive |
| Carnitine acyl transferase 1 | PPARα | Positive |
| Carnitine acyl transferase 2 | PPARα | Positive |
| CYP7A1 | FXR | Negative |
| MTTP | Glucocorticoid | Positive |
| MTTP | PPARα | Positive |
| TG storage | PPARα | Positive |
| DGAT | PPARα | Negative |
| Phosphatidic acid phosphatase | PPARα | Negative |
| LPAAT | PPARα | Negative |
| GPAAT | SREBP1c | Positive |
| Bile acid excretion | LXR-α | Positive |
| SREBP2 | SREBP2 | Positive |
| SREBP-1c | SREBP-1c | Positive |
| SREBP-1c | LXR-α | Positive |
| SREBP-1c | Insulin | Positive |
| SREBP-1c | FXR | Negative |
| SREBP-1c | AMPK | Negative |
| ChREBP | Glucose | Positive |
| FXR | PGC1A | Positive |
| PGC1A | FOXO1 | Positive |
| Cholesterol utilization | Cholesterol | Positive |
| AdipoR1 | FOXO1 | Positive |
| AdipoR1 | Adiponectin | Positive |
| ApoA1 | Cholesterol | Positive |
| CETP | LXR-α | Positive |
| LDLR | SREBP-1c | Positive |
| Lipoprotein lipase | Insulin | Positive |
| Lipoprotein lipase | PPARγ | Positive |
| Lipoprotein lipase | Glucocorticoid | Positive |
| CD36 | PPARα | Positive |
| CD36 | TNFα | Positive |
| HMG CoA Synthase | PPARα | Positive |
| HMG CoA Synthase | Glucagon | Positive |
| HMG CoA Synthase | FOXO1 | Positive |
| HMG CoA Synthase | Insulin | Negative |
| HMG CoA Lyase | PPARα | Positive |
| HMG CoA Lyase | Glucagon | Positive |
| HMG CoA Lyase | Insulin | Negative |
| Stearoyl CoA Desaturase | LXR-α | Positive |
| Stearoyl CoA Desaturase | SREBP-1c | Positive |
| Stearoyl CoA Desaturase | Leptin | Negative |
| Stearoyl CoA Desaturase | Palmitoleate | Negative |
| ATP Citrate Lyase | SREBP2 | Positive |
| ATP Citrate Lyase | Insulin | Positive |
| Acetoacetyl CoA thiolase | SREBP2 | Positive |
| HMG CoA Reductase | SREBP2 | Positive |
| CYP51A1 | SREBP2 | Positive |
| DHCR14 | SREBP2 | Positive |
| DHCR7 | SREBP2 | Positive |
| DHCR24 | SREBP2 | Positive |
| Insulin transporter (pancreas) | Glucose | Positive |
| AdipoR2 | FOXO1 | Positive |
| CD36 (peripheral tissues) | Insulin | Positive |
| CD36 (peripheral tissues) | Adiponectin | Positive |
| Acyl CoA oxidase (peripheral tissues) | Adiponectin | Positive |
| Β-oxidation enzymes (adipose tissue) | PPARγ | Positive |
| GLUT4 (adipose tissue) | TNFα | Negative |
| Insulin receptor (adipose tissue) | TNFα | Negative |
| Acetyl CoA carboxylase (adipose tissue) | SREBP-1c | Positive |
| Fatty acyl CoA synthetase (adipose tissue) | SREBP-1c | Positive |
| Glycerokinase (adipose tissue) | PPARγ | Positive |
| Hormone sensitive lipase (adipose tissue) | Glucocorticoid | Positive |
| Fatty acid export (adipose tissue) | Insulin | Negative |
| LDLR (adipose tissue) | SREBP-1c | Positive |
| TNFα | Adiponectin | Negative |
| TNFα | Glucocorticoid | Negative |
| Adiponectin | PPARγ | Positive |
| Adiponectin | Fatty acid (blood) | Positive |
| Adiponectin | TNFα | Negative |
| Leptin | Glucocorticoid | Positive |
| Leptin | Fatty acids (adipose tissue) | Negative |
| CD36 (macrophage) | PPARγ | Positive |
| PPARγ | TNFα | Negative |
| TNFα (macrophages) | TLR4 | Positive |
