# Supplementary material for: SteatoNet: The First Integrated Human Metabolic Model with Multi-layered Regulation to Investigate Liver-Associated Pathologies
Source: PLoS Comput Biol. 2014 Dec 11;10(12):e1003993. doi: 10.1371/journal.pcbi.1003993 (PMC4263370; doi:10.1371/journal.pcbi.1003993)
Supplement: S7 Table — List of post-translational regulators in SteatoNet, their target proteins and the type of regulatory interaction. (DOCX) [file pcbi.1003993.s008.docx]

**Table S7. List of post-translational regulators in SteatoNet, their target proteins and the type of regulatory interaction.**

| **POST-TRANSLATIONAL TARGET** | **REGULATOR** | **TYPE OF REGULATION** |
| --- | --- | --- |
| Insulin receptor | Insulin | Positive |
| Insulin receptor substrate 1 | Insulin receptor | Positive |
| Insulin receptor substrate 1 | TNFα | Negative |
| GLUT1 | Insulin receptor substrate 1 | Positive |
| AKT | Insulin receptor substrate 1 | Positive |
| GSK3β | AKT | Negative |
| Glycogen synthase | Glucagon | Negative |
| Phospho-6-fructokinase 2 | Protein kinase A | Negative |
| Fructose-1,6-bisphosphatase | Fructose-2,6-bisphosphate | Negative |
| Fructose-2,6-bisphosphatase | Protein kinase A | Positive |
| Phosphofructokinase | Fructose-2,6-bisphosphate | Positive |
| Glucokinase | Glucokinase regulatory protein | Negative |
| Glucokinase regulatory protein | Blood glucose | Negative |
| Glycogen Phosphorylase | Phosphorylase kinase | Positive |
| Phosphorylase kinase | Protein kinase A | Positive |
| Protein kinase A | Glucagon | Positive |
| TNFα (liver) | Blood Fatty acids | Positive |
| TNFα | Adipo R1 | Negative |
| ChREBP | Glucose | Positive |
| Carnitine palmitoylacyltransferase 1(liver) | Malonyl CoA | Negative |
| PPARα | Blood Fatty acids | Positive |
| PPARα | Adipo R1 | Positive |
| Hormone sensitive lipase (liver) | Protein kinase A | Positive |
| SREBP-1c | AMPK | Negative |
| SREBP-1c | Insulin rector substrate 1 | Positive |
| SREBP-1c | Glucose | Positive |
| SREBP-1c | SREBP cleavage activating protein | Positive |
| SREBP cleavage activating protein | Cholesterol | Negative |
| LXRα | Cholesterol | Positive |
| LXRα | D4 Lanosterol | Positive |
| LXRα | FXR | Negative |
| SREBP2 | SREBP cleavage activating protein | Positive |
| SREBP2 | AMPK | Negative |
| PGC1α | Blood Fatty acids | Positive |
| PGC1α | Glucagon | Positive |
| FXR | Bile acids | Positive |
| FXR | PGC1α | Positive |
| FOXO1 | AKT | Negative |
| AMPK | Adipo R1 | Positive |
| Adipo R1 | Adiponectin | Positive |
| Acyl CoA- Cholesterol Acyltransferase | Cholesterol | Positive |
| Acetyl CoA carboxylase | Citrate | Positive |
| Acetyl CoA carboxylase | AMPK | Negative |
| Acetyl CoA carboxylase | Palmitoyl CoA | Negative |
| Pyruvate Carboxylase | Acetyl CoA | Positive |
| Pyruvate kinase | Alanine | Negative |
| Pyruvate kinase | Protein kinase A | Negative |
| Alanine aminotransferase | Alanine | Negative |
| Aspartate aminotransferase | Aspartate | Negative |
| Asparagine synthase | Asparagine | Negative |
| Phosphoglycerate dehydrogenase | Serine | Negative |
| Serine Transhydroxymethylase | Glycine | Negative |
| Methionine adenosyltransferase | Cysteine | Negative |
| Phenylalanine hydroxylase | Tyrosine | Negative |
| Pyyroline-5-Carboxylate Reductase | Proline | Negative |
| HMGCR | Cholesterol | Negative |
| Glucocorticoid receptor | TNFα (liver) | Positive |
| Glucagon release | Glucose | Negative |
| Glucagon release | Insulin | Negative |
| Insulin release | Glucose | Positive |
| Insulin release | Leptin | Negative |
| GLUT4 (tissue) | Insulin receptor (tissue) | Positive |
| GLUT4 (tissue) | AMPK (tissue) | Positive |
| GLUT4 (tissue) | Glucocorticoid receptor | Negative |
| Acetyl CoA carboxylase (tissue) | Fatty acids (tissue) | Negative |
| Acetyl CoA carboxylase (tissue) | AMPK (tissue) | Negative |
| Adipo R2 (tissue) | Adiponectin | Positive |
| AMPK (tissue) | Adipo R2 | Positive |
| FOXO1 (tissue) | AKT (tissue) | Negative |
| AKT (tissue) | Insulin receptor (tissue) | Positive |
| Insulin receptor (tissue) | Insulin | Positive |
| Insulin receptor (tissue) | TNFα (adipose) | Negative |
| Ubiquitination complex | Blood fatty acids | Positive |
| Ubiquitination complex | Insulin receptor (tissue) | Negative |
| CD36 (tissue) | Ubiquitination complex | Negative |
| Fatty acid oxidation (tissue) | Malonyl CoA (tissue) | Negative |
| Insulin receptor (adipose) | Insulin | Positive |
| Insulin receptor (adipose) | TNFα (adipose) | Negative |
| mTOR (adipose) | Insulin receptor (adipose) | Positive |
| SREBP-1c (adipose) | mTOR | Positive |
| Acetyl CoA carboxylase (adipose) | Saturated fatty acids (adipose) | Negative |
| GLUT4 (adipose) | Insulin receptor (adipose) | Positive |
| GLUT4 (adipose) | Glucocorticoid receptor | Negative |
| GLUT4 (adipose) | Leptin | Negative |
| Hormone sensitive lipase (adipose) | TNFα (macrophage) | Positive |
| Hormone sensitive lipase (adipose) | Leptin | Positive |
| Hormone sensitive lipase (adipose) | Protein phosphatase 1(adipose) | Negative |
| Protein phosphatase 1(adipose) | Insulin receptor (adipose) | Positive |
| Protein kinase A (adipose) | Glucagon | Positive |
| Protein kinase A (adipose) | Insulin receptor (adipose) | Negative |
| TNFα (adipose) | Blood fatty acids | Positive |
| Leptin release | Protein kinase A (adipose) | Negative |
| Leptin release | Fatty acids (adipose) | Negative |
| Leptin release | Insulin receptor (adipose) | Positive |
| Leptin release | Glucose (adipose) | Positive |
| Leptin release | TNFα (adipose) | Positive |
| PPARγ | Adiponectin | Positive |
| PPARγ | Blood fatty acids | Positive |
| TLR4 (macrophage) | Blood fatty acids | Positive |
| Lipoprotein lipase | Blood Glucose | Positive |
| Lipoprotein lipase | Insulin receptor (adipose) | Positive |
