# Supplementary material for: SteatoNet: The First Integrated Human Metabolic Model with Multi-layered Regulation to Investigate Liver-Associated Pathologies
Source: PLoS Comput Biol. 2014 Dec 11;10(12):e1003993. doi: 10.1371/journal.pcbi.1003993 (PMC4263370; doi:10.1371/journal.pcbi.1003993)
Supplement: S1 Text — Instructions for accessing SteatoNet on Open Modelica Connnection Editor OMEdit. (DOCX) [file pcbi.1003993.s011.docx]

**Supplementary Text S1. Instructions for accessing SteatoNet on Open Modelica Connection Editor OMEdit.**

- OMEdit can be downloaded for free on the following webpage <https://openmodelica.org/index.php/home/tools/165>
- On installation, load the SysBio library folder (provided as supplementary file) (File>Load Library).
- To load the model, click on File>Open model/library and select the NAFLD_model_flux_sensitivity.mo file. The file will appear in the left-hand tab of the Welcome window. Double click on the file name to load the model in the Modeling window.
- To simulate the model, click on the Simulation tab>Simulation setup. Set the simulation Stop Time and choose the Integration method as ‘dassl’ with a tolerance ≥0.0001.
- Model variable profiles can be viewed in the Plotting window. Variable concentrations are labelled with a suffix of ‘.Q’ e.g. Glucose_liver.Q represents the concentration of hepatic glucose.
- Disturbances have to be triggered by altering parameter values in the Modelling window e.g. massflow values of the external influx source objects (e.g. Glucose_source) or ‘k1’ values for protein objects (k1>0 results in elevated protein degradation, k1<0 results in protein overexpression), prior to simulation and plotting.

The SteatoNet model has been tested on the latest nightly build of Open Modelica (1.9.1+dev) on 64-bit windows7 computer with 8Gb of RAM and intel core vPro i7 processor. It must be highlighted that due to the large size of the model, there is a substantial memory and time requirement to translate and compile the model, in the absence of which software crashes can be expected. Moreover, the presence of bugs in OMEdit, which is being continually developed to a more robust open-access version, may also result in software errors. In comparison, Dymola (version 7.4), which was originally utilized to generate and validate the SteatoNet, has the advantage of being able to handle large systems (>10,000 equations) and hence, can rapidly (within seconds) and stably simulate the SteatoNet. Thus, the Dymola software provides the best and most stable simulation conditions for SteatoNet implementation.
